# Cu_A_-based chimeric T1 copper sites allow for independent modulation of reorganization energy and reduction potential†
†Electronic supplementary information (ESI) available: Resonance Raman spectra, electrochemical data and computational (MDS and QM/MM) results. See DOI: 10.1039/d0sc01620a


**DOI:** 10.1039/d0sc01620a

**Published:** 2020-06-01

**Authors:** Jonathan Szuster, Ulises A. Zitare, María A. Castro, Alcides J. Leguto, Marcos N. Morgada, Alejandro J. Vila, Daniel H. Murgida

**Affiliations:** a Instituto de Química Física de los Materiales, Medio Ambiente y Energía (INQUIMAE, CONICET-UBA) , Argentina . Email: dhmurgida@qi.fcen.uba.ar; b Departamento de Química Inorgánica, Analítica y Química-Física , Facultad de Ciencias Exactas y Naturales , Universidad de Buenos Aires , Buenos Aires , Argentina; c Instituto de Biología Molecular y Celular de Rosario (IBR, CONICET-UNR) , Argentina; d Departamento de Química Biológica , Facultad de Ciencias Bioquímicas y Farmacéuticas , Universidad Nacional de Rosario , Rosario , Argentina

## Abstract

Attaining rational modulation of thermodynamic and kinetic redox parameters of metalloproteins is a key milestone towards the (re)design of proteins with new or improved redox functions. Here we report that implantation of ligand loops from natural T1 proteins into the scaffold of a Cu_A_ protein leads to a series of distorted T1-like sites that allow for independent modulation of reduction potentials (*E*°′) and electron transfer reorganization energies (*λ*). On the one hand *E*°′ values could be fine-tuned over 120 mV without affecting *λ*. On the other, *λ* values could be modulated by more than a factor of two while affecting *E*°′ only by a few millivolts. These results are in sharp contrast to previous studies that used T1 cupredoxin folds, thus highlighting the importance of the protein scaffold in determining such parameters.

## Abstract 2

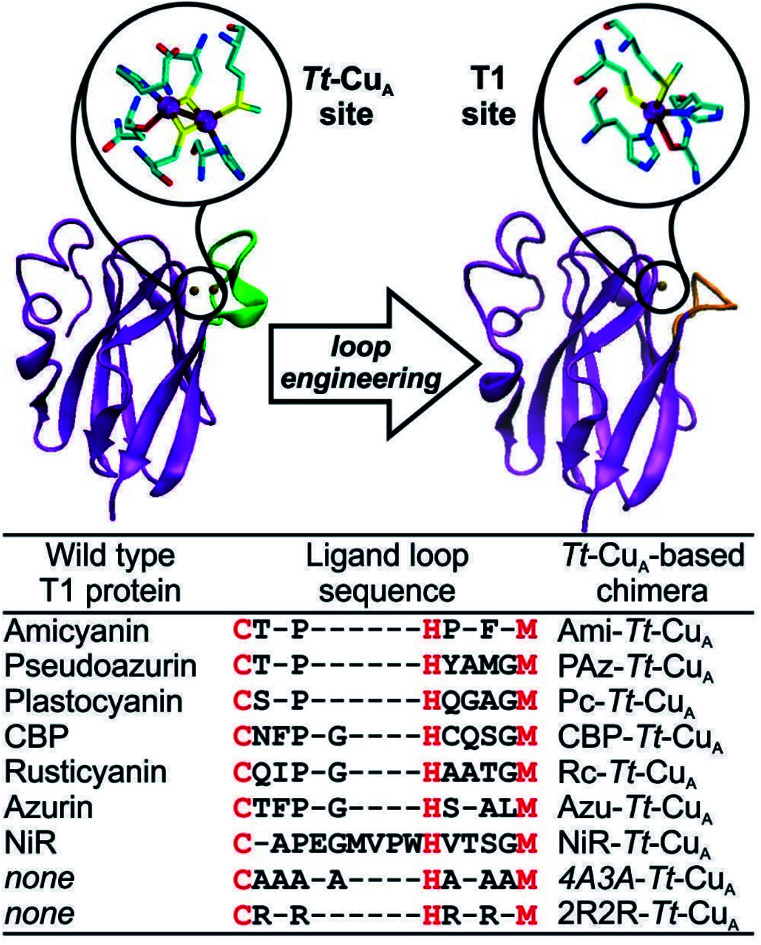
Attaining rational modulation of thermodynamic and kinetic redox parameters of metalloproteins is a key milestone towards the (re)design of proteins with new or improved redox functions.

## Introduction

Redox metalloproteins are ubiquitous in nature and are implicated in a broad range of catalytic and electron transfer (ET) functions that impose quite diverse thermodynamic and kinetic requirements to the redox sites.^1^ Understanding how natural evolution conjugates structural and/or dynamical features to modulate redox parameters is essential to envisage a successful metalloprotein *de novo* design or redesign.^2^

In the case of copper redox proteins, binuclear Cu_A_ and mononuclear T1 sites are implicated in ET reactions, while mononuclear T2 centers may also have catalytic activity.^3^ The first two types of centers share the cupredoxin fold and the interesting feature that all but one of the coordinating amino acids are located in a single loop that connects two β-strands.^1^,^4^ Therefore, along with point mutations, replacement of the entire ligand loop by sequences from other proteins or unnatural sequences,^5^ has become one of the preferred strategies for modulating the electronic properties of T1 4,6–23 and Cu_A_ sites.^24^–^30^ This methodology has also allowed for the successful insertion of Cu_A_ sites into the scaffold of T1 31–35 proteins and *vice versa*.^36^

So far, most efforts have focused on tuning reduction potentials (*E*°′) of T1-like mononuclear centers through first and second sphere perturbations, attaining up to 700 mV modulation.^1^,^15^ The tuning of kinetic ET parameters such as the reorganization energy (*λ*) received significantly less attention for metalloproteins in general and for T1 sites in particular,^21^,^37^ and no clear patterns have been established for the simultaneous or independent modulation of *λ* and *E*°′.

Here we report the functional characterization of a series of distorted T1 chimeric proteins that were obtained by engineering of the ligand loop of the Cu_A_ site from *Thermus thermophilus ba*_3_ cytochrome *c* oxidase. We show that this strategy allows for the independent modulation of *λ* and *E*°′ through the sequence and length of the ligand loop, while preserving the native T1 ligand set. The key for attaining this tunability is the use of a scaffold not evolutionary optimized for harboring T1 sites that, therefore, differs in flexibility, geometrical constrains and solvent accessibility to the site cavity. This approach, which has not been sufficiently explored in the past, may contribute to expanding the current tool-box for metallo-protein redesign.

## Results and discussion

**Fig. 1:**
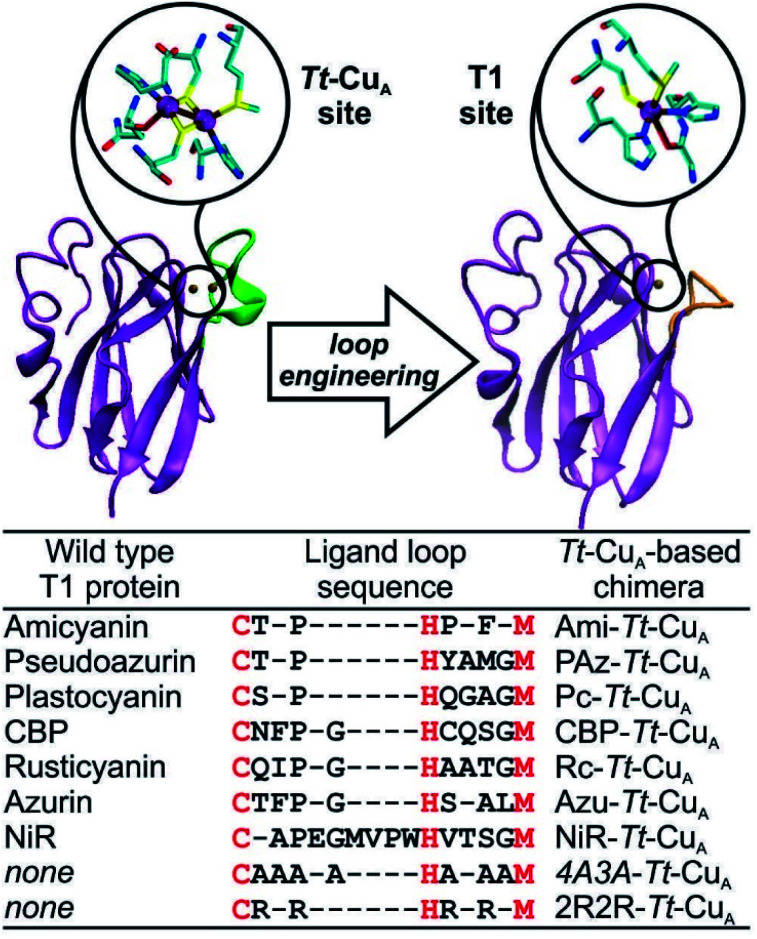
X-ray crystallographic structures of native *Tt*–Cu_A_- (PDB ID ; 2CUA)^38^ on the left, and that of Ami–*Tt*–Cu_A_ on the right (PDB ID ; 5U7N).^36^ The loop replaced in the chimeras is indicated in green and orange, respectively. The loop sequences employed to obtain the different chimeras are listed below. Red letters denote the conserved ligand set.

Type 1-like copper sites were engineered into the scaffold of the Cu_A_-containing soluble domain of the *ba*_3_ oxygen-reductase from *Thermus thermophilus* (*Tt*–Cu_A_).^38^ Specifically, the sequence of the loop that carries five of the six ligands in the Cu_A_ site was replaced by ligand loop sequences of T1 copper proteins from seven different organisms and two artificial sequences (Fig. 1) following established procedures.^36^ The sequences were chosen aiming to cover a wide range of geometric distortions, from classic axial blue sites, such as azurin and amicyanin, to strongly perturbed rhombic green sites, such as pseudoazurin, cucumber basic protein and nitrite reductase. Thus, the ligand loops of the chimeras differ in length and sequence, but contain a preserved Cys/His/Met T1 ligand set, which is completed with His75 from the *Tt*–Cu_A_ scaffold.

### Spectroscopy and structural modelling

UV-vis spectra of mononuclear copper proteins are well established reporters of the geometric distortions of the metal sites.^39^,^40^ Canonical axial T1 sites are characterized by an intense absorption at around 600 nm (*ε*_max_ ≈ 3000–6000 M^–1^ cm^–1^) that accounts for the typical blue colour. Rhombic sites, in contrast, present an intense feature around 450 nm (*ε*_max_ ≈ 1500–3500 M^–1^ cm^–1^) responsible for the greenish hue, along with a much weaker 600 nm band compared to axial sites.^41^,^42^ Both bands have been assigned to S_cys_ → Cu^2+^ ligand to metal charge transfer (LMCT) transitions, and their relative intensities were rationalized by the so-called coupled distortion model in terms of differential overlap between the cysteine-3p and copper-3d_*x*^2^–*y*^2^_ orbitals. Gradual conversion from a typical blue site into a perturbed green site has been associated with a shortening of the Cu–S_Met_ distance and concomitant lengthening of the Cu–S_Cys_ bond that results in a degree of tetragonal distortion and correlates with the intensity ratio of the two bands, *i.e.* with *ε*_450_/*ε*_600_.^39^

**Fig. 2:**
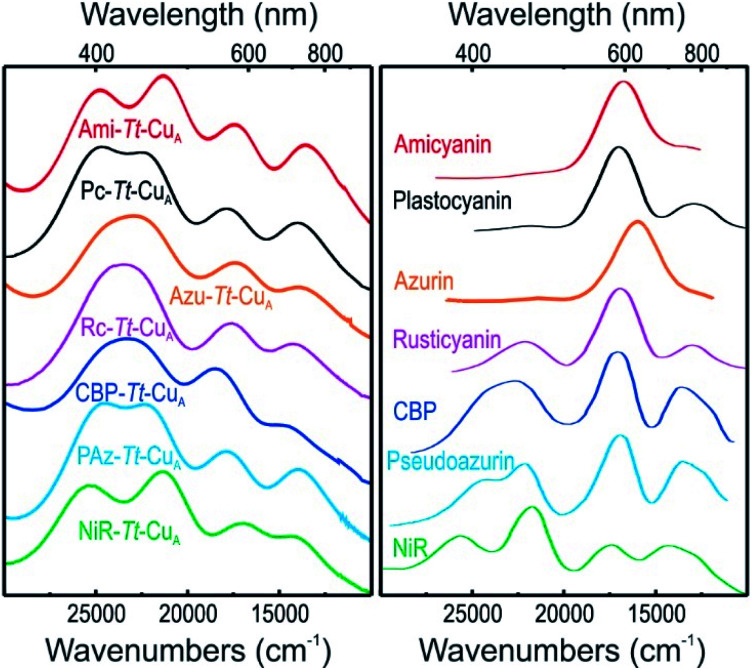
(Left) Electronic absorption spectra of the chimeras engineered in the *Tt*–Cu_A_ scaffold. (Right) Electronic absorption spectra of wild type proteins that harbor T1 copper sites taken from literature.^40^,^41^,^44^–^47^ Spectra of the 2R2R–*Tt*–Cu_A_ and ; 4A3A–*Tt*–Cu_A_ variants, as well as spectral deconvolutions, are shown in Fig. S1.†

The absorption spectra of the chimeras are displayed in Fig. S1† as well as in Fig. 2 along with those of the corresponding native T1 proteins. The spectra of the native proteins (Fig. 2, right panel) are ordered from top to bottom based on the distortion level. Note that the degree of spectral distortion in the left panel does not follow the same trend. In contrast, the absorption spectra of all the chimeras display two partially overlapping strong bands at around 400–450 nm that are assigned to S_Met_ → Cu^2+^ and pseudo-*σ* S_Cys_ → Cu^2+^ LMCT transitions.^36^ The *ε*_450_/*ε*_600_ ratios of the chimeras range from 1.10 to 1.64 (Table S1 and Fig. S1†), thus approaching the value reported for nitrite reductase, 1.74,^42^ which is the most perturbed T1 site reported to date. Thus, the UV-vis spectra are consistent with strengthened Cu–S_Met_ interactions within the *Tt*–Cu_A_ scaffold. Unfortunately, all attempts to obtain high quality crystals for X-ray diffraction were unsuccessful except for the recently reported Ami–*Tt*–Cu_A_ chimera (pdb ; 5U7N).^36^ In agreement with the UV-vis spectra, this variant has a Cu–S_Met_ distance of only 2.35 Å, *i.e.* significantly shorter than in native tetragonally distorted sites such as pseudoazurin^43^ and nitrite reductase,^44^ which have values of 2.75 Å and 2.55 Å, respectively.

**Fig. 3:**
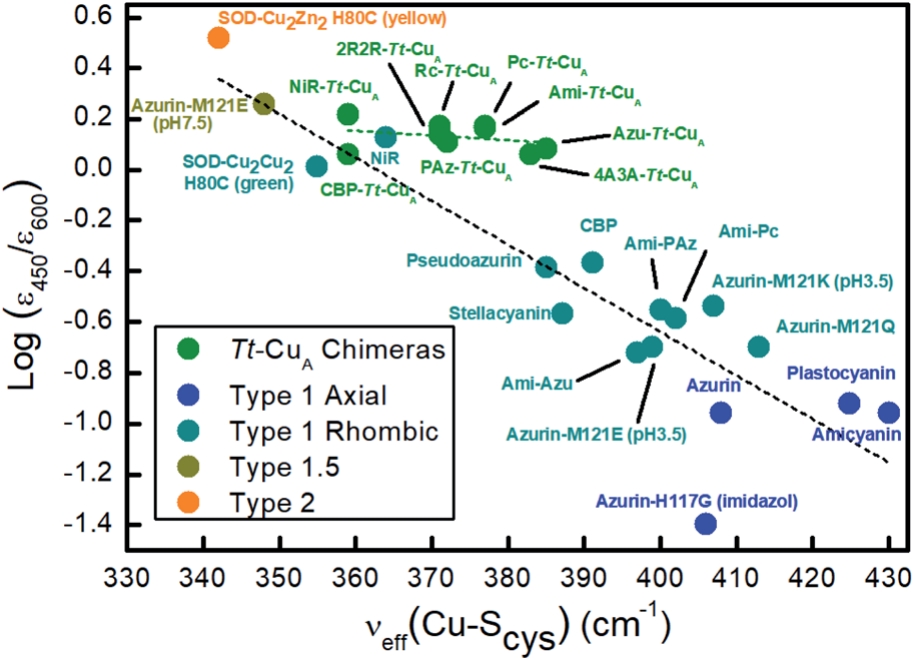
Correlation of the *ε*_450_/*ε*_600_ ratio with the Cu–S_Cys_ effective stretching frequency for different types of mono-copper sites. Green symbols correspond to the *Tt*–Cu_A_ chimeras from this work. The rest of the symbols are data taken from literature^5^,^52^,^55^ for different types of mononuclear Cu centers, as indicated in the inset, and include axial and rhombic T1 centers, one T2 site and one so-called T1.5 center of geometry intermediate between T1 and T2 sites.^52^ Dashed lines are included to guide the eye.

Resonance Raman (rR) spectra of the chimeras display the typical features of mononuclear copper sites in the 330–430 cm^–1^ region (Fig. S2†), assigned to vibrational modes composed of deformations of the cysteine ligand coupled to Cu–S_Cys_ stretching.^45^–^51^ The effective vibrational frequencies, *ν*Cu–Cyseff, calculated as the intensity-weighted average of all the rR signals,^49^,^50^,^52^ vary between 359 and 385 cm^–1^ (Table S1†) and differ significantly from those of the wild type proteins containing the same loop sequences, consistent with the differences observed in UV-vis absorption spectroscopy. The *ν*Cu–Cyseff values follow qualitatively the same type of trend with the *ε*_450_/*ε*_600_ ratio verified for other natural and engineered mononuclear copper proteins, albeit with significantly smaller slope (Fig. 3), and are in agreement with the description of the engineered centers as T1 sites with a rhombic distortion. The smaller variation of the *ε*_450_/*ε*_600_ ratio observed for the green chimeras compared to other T1 sites suggests that Cu–S_Cys_ distances are relatively similar for all members of this group of proteins, even though *ν*Cu–Cyseff values exhibit larger variability. To further assess the geometrical parameters of the engineered proteins we produced them *in silico* by replacing the corresponding ligand loop sequences into the crystal structure of Ami–*Tt*–Cu_A_ (PDB ID ; 5U7N)^36^ followed by MD and QM/MM calculations. The most relevant structural parameters are summarized in Table S2.† The ligand loop backbones of the model structures are superimposable with those of the corresponding T1 native proteins (Fig. S3†), thus indicating that the loop fold is not significantly influenced by the scaffold to which it is attached, in agreement with previous observations.^16^,^36^ Sidechains, however, may still be affected by steric hindrance imposed by the β-barrel scaffold. The coordinating Cys and Met residues connect a β-sheet and the ligand loop, while the His75 ligand is buried within the β-barrel fold. Steric clashes can be directly transmitted to the first coordination shell and distort the geometry of the metal site. The level of distortion can be quantified through the ratio of Cu–S_Met_ and Cu–S_Cys_ distances, dCu–S_Met_/dCu–S_Cys,_ or, alternatively, through the parameter *τ*_4_ = (360° – (*α* + *β*))/(141°) introduced by Yang *et al.*^53^ Here *α* and *β* are the two largest bond angles, such that *τ*_4_ ranges from 1 for a perfect tetrahedral geometry to 0 for a perfect square planar one. As summarized in Table S3 and Fig. S4,† except for the ; 4A3A–*Tt*–Cu_A_ variant, the calculated dCu–S_Met_/dCu–S_Cys_ and *τ*_4_ parameters of the oxidized chimeras tend to increase with *ε*_450_/*ε*_600_, thus paralleling the experimental trend in terms of distortion. Moreover, absorption spectra obtained from single point calculations reproduce experimental ones reasonably well (Fig. S5†), thereby validating the computational approach. This match is worst for ; 4A3A–*Tt*–Cu_A_, thus confirming the poorer predictive capability of this particular structural model that, therefore, is excluded from subsequent analysis. For the remaining proteins the calculations predict a partial loss of rack effect compared to natural cupredoxins,^54^ as average *τ*_4_ values for the oxidized and reduced species are 0.67 and 0.92, respectively (Δ*τ*_4_ = 0.25; Table S3†). While the oxidized forms are more tetragonally distorted towards square-planar, the geometry of the reduced metal sites are more tetrahedral (Fig. S6 and Table S2†). The calculations show redox-state-dependent reorientations of the three ligands belonging to the engineered loop and of the fourth ligand His75 from the native *Tt*–Cu_A_. For comparison, Δ*τ*_4_ values previously estimated for native plastocyanin and its protein-free T1 center are 0.12 and 0.34, respectively.^55^

Interestingly, while the set of atoms S_Met_–Cu–S_Cys_–Cβ_Cys_–Cα_Cys_–N_Cys_ is invariably coplanar in native proteins and rarely deviates more than a few degrees from 180° or 0°,^47^ the calculations indicate that this planarity is lost in the chimeras (Table S2†). This prediction is consistent with the weakness of their rR signals compared to the wild type proteins, as coplanarity is essential for rR enhancement *via* kinematic vibronic coupling.^49^ The broken planarity also implies normal mode redistribution and, therefore, *ν*Cu–Cyseff is not expected to provide straightforward structural information based on rules derived for coplanar systems.

Taken together, the obtained results indicate that loop engineering of mononuclear copper sites into the *Tt*–Cu_A_ scaffold leads to novel T1 centers with unique distortions imposed by the protein matrix. The loop sequence appears to have a relatively subtle role in modulating the geometric and electronic structures of the engineered sites. These conclusions are qualitatively in good agreement with previous observations on different chimeric systems.^6^,^11^,^14^,^56^ For instance, replacement of the ligand loop of amicyanin by the sequences of the distorted rhombic pseudoazurin and nitrite reductase results in chimeras with absorption spectra that closely resemble wild type amicyanin.^6^ Analogously, introduction of the amicyanin ligand loop into the pseudoazurin fold leads to spectral features similar to native pseudoazurin.^11^

### Modulation of reduction potentials

**Fig. 4:**
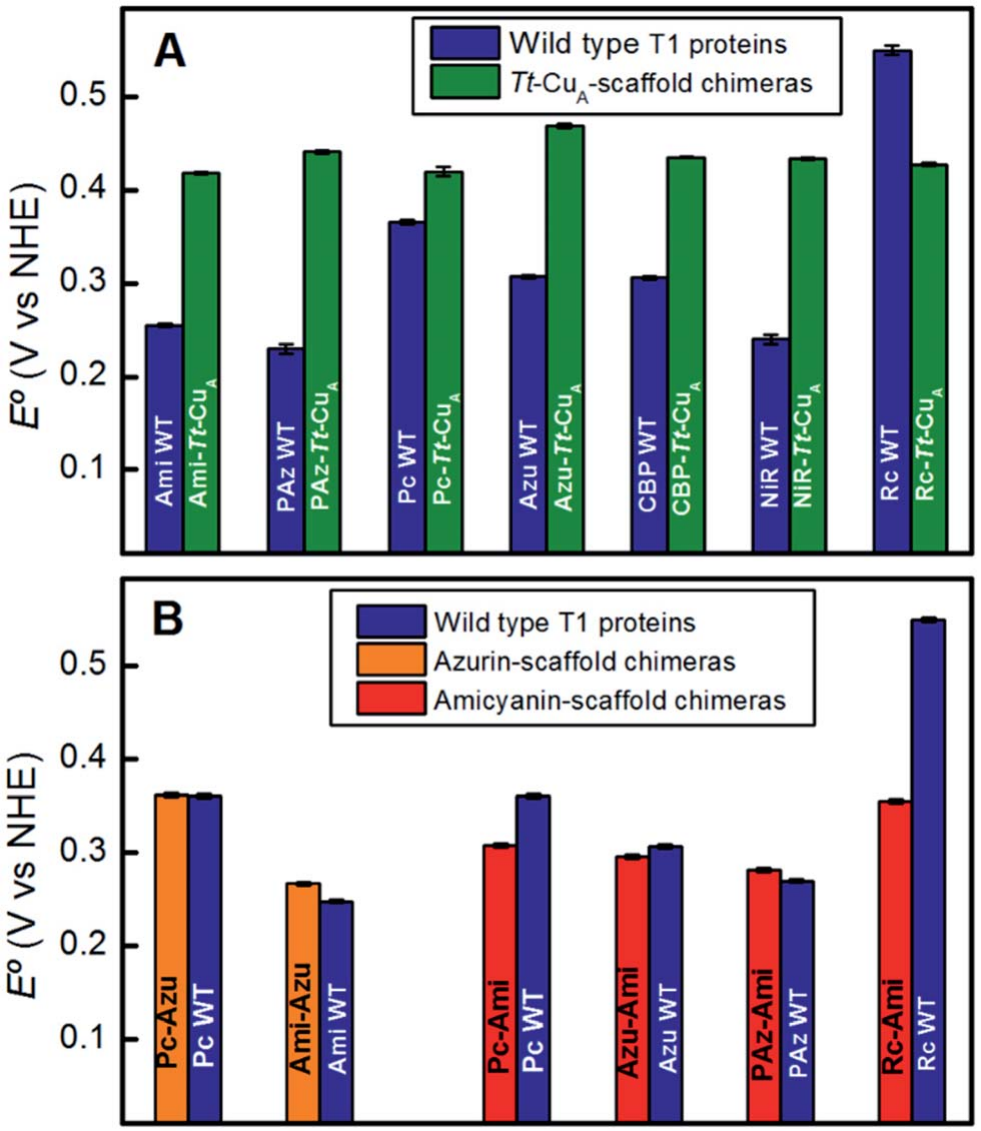
(A) Reduction potentials of the *Tt*–Cu_A_ chimeras compared with the corresponding native T1 sites. (B) Reduction potentials of chimeras based on the azurin and amicyanin scaffolds compared to the corresponding native sites. Except for *Tt*–Cu_A_ chimeras, values are taken from literature.^5^,^13^,^45^

To assess the functional features of the different chimeras we performed cyclic voltammetry (CV) experiments in solution (Fig. S7†). The CVs obtained for all protein variants are characterized by peak-to-peak separations of around 60 mV, estimated charge transfer coefficients of 0.5 and peak currents that scale with the square root of the scan rate, thus indicating diffusion-controlled one-electron reversible redox processes (Fig. S8†). With the only exception of Rc–*Tt*–Cu_A_, all the proteins exhibit reduction potentials (*E*°′) well above those of the corresponding native T1 proteins (Fig. 4A and Table S1†). The relatively large magnitude of the shifts, which is in the range of 50–210 mV, is a distinct feature of these chimeras that contrasts with previous results obtained by loop engineering of natural T1 proteins^6^,^11^,^14^,^57^–^60^ (Fig. 4B). These shifts can be partially ascribed to the weakening of the rack effect in the chimeras. In agreement with this interpretation, partial denaturation of *Pseudomonas aeruginosa's* azurin was reported to result in 130 mV upshift of *E*°′.^61^–^63^ In addition, the distance and orientation of the backbone carbonyl belonging to the residue located in the axial position (Fig. S9 and Table S2†) may also play a role.^44^,^54^,^64^–^74^ For the chimeras presented in this work these distances are around 4.5–4.7 Å, which are either lower than in the corresponding native T1 centers, such as for Rc–*Tt*–Cu_A_, or higher, as for the rest of the chimeras with natural loop sequences, thus resulting in downshifts or upshifts of *E*°′, respectively (Fig. S10†). Finally, the length of the H-bond from Gly76 backbone to the sulfur atom of the coordinating Cys110, which influences the relative stabilization of Cu^1+^*vs.* Cu^2+^ through the electron density of the copper–sulfur bond,^64^,^75^,^76^ is largely constant for the set of chimeras (Fig. S9 and Table S2†) with an average value of 3.6(±0.1) Å. These structural elements, and possibly others, are likely to determine the *E*°′ shift of the chimeras with respect to the corresponding native T1 proteins.

**Fig. 5:**
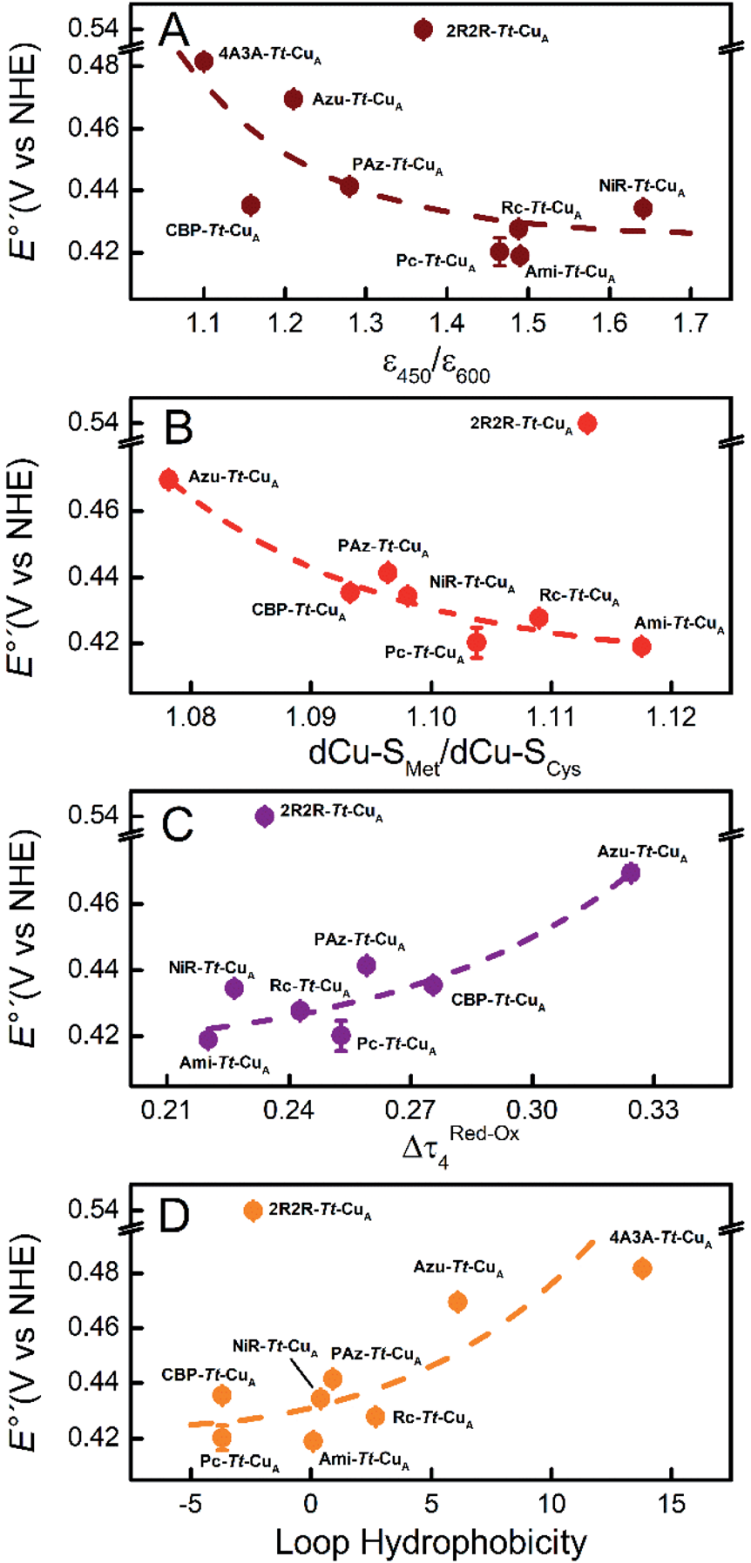
Reduction potentials of the *Tt*–Cu_A_ chimeras as function of (A) the experimental *ε*_450_/*ε*_600_ ratio, (B) the calculated dCu–S_Met_/dCu–S_Cys_ ratio, (C) the calculated Δ*τ*_4_ = *τ*_4_ (red) – *τ*_4_ (ox) parameter and (D) the hydrophobicity of the ligand loop. Dashed lines are included to guide the eye. Error bars represent the standard deviation of no less than 3 independent measures.

Comparison of the different *Tt*–Cu_A_-based chimeras with each other provides some clues to understanding the fine tuning of *E*°′. Albeit with some dispersion, *E*°′ clearly decreases with increasing *ε*_450_/*ε*_600_ and dCu–S_Met_/dCu–S_Cys_ ratios (Fig. 5A and B), *i.e.* with the distortion of the oxidized sites. This variation is consistent with strengthened copper–methionine interactions in the chimeras relative to the native T1 centers, thus suggesting that scaffold-induced perturbations readily translate to the metal site and affect the Cu^2+^/Cu^1+^ relative stabilities. In line with these conclusions, we observe a clear dependency of *E*°′ with Δ*τ*_4_ for the series of chimeras (Fig. 5C). In addition, *E*°′ values increase with the hydrophobicity of the ligand loop (Fig. 5D), which can be rationalized in terms of destabilization of Cu^2+^ relative to Cu^1+^ by increasingly hydrophobic environments.^13^,^17^,^28^,^77^–^79^ Note that 2R2R–*Tt*–Cu_A_ is the only variant that strongly deviates from all the correlations shown in Fig. 5 and has by far the highest *E*°′ of the series (540 mV; Table S1†). This value is 120 mV higher than for Ami–*Tt*–Cu_A_, which has the same loop length but different sequence (Fig. 1) and very similar *ε*_450_/*ε*_600_, dCu–S_Met_/dCu–S_Cys_, *τ*_4_ and Δ*τ*_4_ values (Tables S1 and S3†). We ascribe this additional shift to the fact that four out of seven residues of the ligand loop are positively charged arginines (pH = 7.0). The rest of the chimeras contain only neutral amino acids, with the only exception of NiR–*Tt*–Cu_A_ that contains one glutamic acid in the 15-residue long loop and has one of the lowest *E*°′ values. Most likely the high density of positive charges in the short loop of 2R2R–*Tt*–Cu_A_ strongly estabilizes Cu^2+^*versus* Cu^1+^, overwhelming the effect of subtle geometrical distortions.

The solvent accessible surface area (SASA) of the metal site (copper ion plus side chains of the coordinating residue) is within the range of 440–480 Å^2^ for all the chimeras (Table S2†), which is consistent with the finding that these proteins are able to bind small exogenous ligands.^36^ This parameter varies up to 5% with the loop length and redox state (Fig. S11†). However, experimentally determined E°′ values show no clear correlations with neither absolute SASA values, nor with SASA differences between oxidized and reduced states (Fig. S12†), thus indicating that the small variations in solvent accessibility do not significantly contribute to the modulation of *E*°′. Similar observations have been rationalized in the past in terms of enthalpy/entropy compensation effects.^80^

In summary, the evidence suggests that, within the series of chimeras, *E*°′ can be tuned over 120 mV through at least three variables: geometrical distortions of the metal site, hydrophobicity of the ligand loop and local charges. The first two account for small variations of *E*°′ that for natural loop sequences are of up to 50 mV. In contrast, replacement of the ligand loop in natural T1 proteins leads to either upshifts or downshifts of up to *ca.* 100 mV of *E*°′ with respect to the wild type scaffold-carrying protein to match that of the wild type loop-carrying variant.^6^,^14^,^56^ For the chimeras based on the *Tt*–Cu_A_ scaffold this transfer of information is not verified. As we will show in the following sections, the small variation of *E*°′ observed for *Tt*–Cu_A_-based chimeras with natural loop sequences represents a unique opportunity to independently modulate *E*°′ and other relevant electron transfer parameters, such as reorganization energies, that may be more strongly dependent on loop architecture.

### Modulation of reorganization energies

**Fig. 6:**
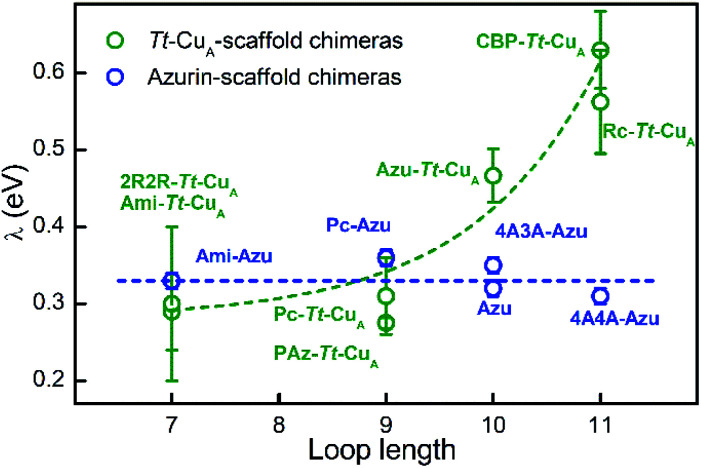
Reorganization energies of *Tt*–Cu_A_-based chimeras (green; this work) and azurin-based chimeras (blue; taken from Monari *et al.*^85^) as a function of the length of the ligand loop. Dashed curves are included to guide the eye. Error bars represent the standard deviation of no less than 3 independent measures.

In terms of Marcus semiclassical theory,^81^ electron transfer rates are determined by the protein intrinsic parameters *E*°′ and reorganization energy (*λ*), in addition to the donor–acceptor electronic coupling. To assess the influence of the *Tt*–Cu_A_-scaffold and of the ligand loop architecture on *λ* we performed protein film voltammetry (PFV) experiments with the different chimeras adsorbed on Au electrodes coated with self-assembled monolayers (SAMs) of HS–(CH_2_)_15_–CH_2_OH and HS–(CH_2_)_15_–CH_3_ in 3 : 2 ratios. This SAM composition has been shown to provide a suitable interface for adsorption and direct electrochemistry of cupredoxins in the nonadiabatic regime with retention of the active site structure.^29^,^82^ Except for ; 4A3A–*Tt*–Cu_A_ and NiR–*Tt*–Cu_A_ that gave no electrochemical signals, the voltammetries of the adsorbed chimeras yield quasi-reversible responses with charge transfer coefficients between 0.4 and 0.5, and peak currents that scale linearly with the scan rates, as expected for surface-confined redox active species (Fig. S13–S15†). Furthermore, the reduction potentials are very similar to those obtained for the proteins in solution (Table S4†), thereby confirming the structural integrity of the adsorbed chimeras. Heterogeneous electron transfer rate constants, *k*0ET, were determined from the peak-to-peak separation of the voltammograms as function of the scan rates according to Laviron's formalism (Fig. S16 and S17†).^83^ The reorganization energies were estimated from the temperature dependence of *k*0ET in the range 4–40 °C treating the data in terms of Arrhenius equation and assuming *E*_a_ ≈ Δ*G*^#^ ≈ *λ*/4 (Fig. S18†). As control experiments, *λ* values were also determined at constant temperature by fitting trumpet plots obtained over a broad range of scan rates with Marcus expression for heterogeneous ET on metal electrodes (Tables S4 and S20†).^84^ In spite of the larger uncertainty of the second method, *λ* values obtained with the two approaches are essentially identical (Fig. S19†). As shown in Fig. 6, and in sharp contrast to reduction potentials, *λ* values vary strongly between chimeras by up to a factor of 2.3. Moreover, in opposition to previous constructs based on the azurin scaffold,^85^*Tt*–Cu_A_-based chimeras show a clear correlation of *λ* with the length of the engineered ligand loop. Notably, the Ami–*Tt*–Cu_A_ and 2R2R–*Tt*–Cu_A_ variants differ strongly in terms of *E*°′ but have identical *λ* as they share the same loop length.

To the best of our knowledge, this is the first time that such a strong modulation of *λ* is achieved by means of loop exchange with preservation of the T1 ligand set.

The rise of *λ* with the loop length is paralleled by a similarly strong increase with the SASA values calculated for the reduced proteins (Fig. S21†), in agreement with previous reports on related systems.^37^,^86^,^87^ Albeit with larger scattering, *λ* also tends to increase with calculated SASA of the oxidized proteins. The larger scattering reflects redox-state-dependent variations of SASA of up to 6%. These results indicate that the shielding of the metal site in the chimeras becomes less effective the longer is the implanted loop. In addition, they strongly suggest that the variation of *λ* can be largely ascribed to the outer sphere reorganization (*λ*_out_)^81^ of the solvent and, possibly, of the ligand loop. In agreement with this conclusion, experimentally determined *λ* values show no correlation with descriptors of the metal site geometry such as *ε*_450_/*ε*_600_, dCu–S_Met_/dCu–S_Cys_, *τ*_4_ and Δ*τ*_4_ (Fig. S22†). Thus, in spite of the partial loss of rack effect that affects *E*°′, the inner sphere reorganization (*λ*_in_) appears to be a relatively small fraction of *λ* which, instead, is largely determined by *λ*_out_, in line with computational estimates for different metalloproteins.^87^–^89^

## Conclusions

Replacement of the ligand loop of binuclear *Tt*–Cu_A_ by the corresponding sequences from mononuclear cupredoxins yields distorted mononuclear T1 sites that, unlike previously reported T1-like chimeras, allow for independent tuning of crucial thermodynamic and kinetic electron transfer parameters, such as *E*°′ and *λ*. It is shown that *λ* can be more than doubled without affecting *E*°′ by more than a few millivolts that represent less than 5% variation, while *E*°′ can be fine-tuned over 120 mV without affecting *λ*. This peculiar feature is ascribed to the different constraints imposed by the *Tt*–Cu_A_ scaffold compared to mononuclear cupredoxins, as they are optimized to host two and one copper ions respectively. While the backbone structure of the implanted loops is not affected by the *Tt*–Cu_A_ scaffold, the geometry of the metal sites shows small but significant variations that correlate with the shifts of *E*°′. Loop hydrophobicity and local charges are also found to contribute to *E*°′ modulation.

The distortions of the metal sites are redox-state dependent, thus revealing partial loss of the characteristic rack effect. This, however, has no impact on experimentally determined *λ* values. The evidence suggests that this magnitude is largely dominated by the *λ*_out_ contribution. Indeed, *λ* increases strongly with the loop length (not with the sequence) and with solvent accessibility to the metal site, but is independent of the inner sphere reorganization descriptor Δ*τ*_4_.

These results deepen the current understanding of the interplay of thermodynamic and kinetic redox parameters in metalloproteins and their structural determinants. Furthermore, they highlight the key role of the protein scaffold in determining relevant redox parameters of chimeric constructs, thus contributing to expand the current tool-box for metalloprotein design.

## Methods

### Protein preparation

All chimeras were prepared and purified as described previously^36^ and stored in 100 mM phosphate buffer (pH 6.0; 100 mM KCl). Protein samples were buffer exchanged before use by thorough filtration with Amicon Ultracel-10 K filters employing a refrigerated centrifuge at 3800 rpm and 4 °C.

### Electrochemistry

All experiments were performed with either a Gamry REF600 or a PAR263A workstation. Electrochemical cells were placed inside a Faraday cage (Vista Shield) and equipped with a *ca.* 2 mm^2^ homemade polycrystalline gold bead working electrode, a Pt wire auxiliary electrode and an Ag/AgCl (3 M KCl) reference electrode, as well as a circulation thermostat (Lauda Alpha RA8). All potentials in this work are quoted *versus* NHE. Before use Au electrodes were treated as described previously.^82^ Briefly, after thorough chemical and electrochemical treatment, electrodes were incubated overnight in ethanolic solutions containing the desired alkanethiols to form self-assembled monolayer (SAM) coatings. After SAM-coating, electrodes were cycled repeatedly at 0.1 V s^–1^ within the potential windows appropriate for each protein in the measuring electrolyte solution (10 mM HEPES buffer, pH 7.0, containing 250 mM KNO_3_).

For cyclic voltammetry (CV) measurements in solution electrodes were coated with HS–(CH_2_)_6_–OH to prevent protein adsorption and placed into a home-made water jacketed non-isothermal cell that requires *ca.* 40 μL samples with concentrations around 100 μM (10 mM buffer HEPES, pH 7.0, 500 mM KNO_3_). For protein film voltammetry (PFV) experiments electrodes were incubated in 2 mM HS–(CH_2_)_15_–CH_3_/3 mM HS–(CH_2_)_15_–CH_2_OH mixtures to form SAMs, then incubated for 2 hours in 0.1–0.5 mM protein solutions for adsorption and finally transferred to a water-jacketed Gamry-Dr. Bob's cell. PFV's were typically acquired at scan rates between 50 and 500 mV s^–1^.

### Spectroscopy

UV-vis absorption spectra were acquired at 25 °C with a Thermo Scientific Evolution Array spectrophotometer. For resonance Raman (rR) measurements *ca.* 10 μL protein samples were placed in a Linkam THMS 300 thermostat and frozen at 77 K. The spectra were collected in backscattering geometry with a LabRam HR Evolution Raman microspectrometer set at 0.4 cm^–1^ resolution and using either 532 nm or 633 nm excitation. Spectroscopic and electrochemical determinations were performed with the same buffer (10 mM buffer HEPES, pH 7.0).

### Computational methods

Initial models were built from PDB ID code 5U7N for Ami–*Tt*–Cu_A_. Loop variants were built *in silico* through kinematic closure loop modeling using Rosetta.^90^ All structures were relaxed following an equilibration process that consisted of an energy minimization step followed by slow heating from 0 K to 300 K (400 ps). Afterwards, 50 ns long production MD simulations in explicit water were performed at 1 atm and 300 K using the Berendsen barostat and thermostat, respectively. Periodic boundary conditions and Ewald sums were used for long-range electrostatic interactions and a 12 Å cut-off was considered for computing direct interactions. The SHAKE algorithm was used to keep bonds involving hydrogen atoms at their equilibrium length. All simulations were performed with the GPU implementation of the PMEMD module of the AMBER16 package.^91^ The Amber ff14SB force field was used for all standard residues and the Cu site parameters were developed using the MCPB.py builder in AmberTools17.^92^ Cu parameters were obtained for both oxidized and reduce Ami–*Tt*–Cu_A_ and were afterwards used for all the variants. Snapshots of each system were slowly cooled to 0 K (200 ps) in order to obtain the initial structures for QM/MM simulations. These were performed at the DFT level using the SIESTA code with the QM/MM implementation Hybrid.^93^ Basis sets of double zeta plus polarization quality were employed for all atoms with a cut-off and energy shift values of 150 Ry and 25 meV respectively. Calculations were performed under the spin-unrestricted approximation using the generalized gradient approximation functional proposed by Perdew, Burke, and Ernzerhof (PBE).^94^ The scaled position link atom method was used to treat the interface between the QM and MM sections. The QM section included the copper atom and the side chain of the amino acids directly coordinated to Cu. The rest of the protein and water molecules were treated classically using the Amber force field. All atoms included in the MD simulation were included in the QM/MM system and geometry optimization was performed at the QM/MM level for all proteins in the oxidized and reduced states. UV-vis spectra were simulated performing time dependent DFT calculations on the previously optimized QM section, obtaining the energies and intensities of the 50 lowest energy electronic transitions using Gaussian09.^95^ A mixed triple-zeta/double zeta (TZVP) basis set was used for Cu and S atoms, while the 6-31G* basis set was used on all the other atoms. Atom contributions to molecular orbitals and UV-vis spectra were computed with the software Chemissian. Root mean square fluctuations (RMSF) of backbone, solvent accessible surface area (SASA) of Cu and its first coordination and hydrogen bonds were computed with the default settings of the cpptraj module of AmberTools17 for all snapshots.

## Conflicts of interest

There are no conflicts to declare.

## Supplementary Material

Supplementary information
