# Supplementary material for: CuA-based chimeric T1 copper sites allow for independent modulation of reorganization energy and reduction potential
Source: Chem Sci. 2020 Jun 1;11(24):6193–201. doi: 10.1039/d0sc01620a (PMC7480511; doi:10.1039/d0sc01620a)
Supplement: Supplementary file 1 [file SC-011-D0SC01620A-s001.pdf]

Electronic Supplementary Material (ESI) for Chemical Science.
This journal is © The Royal Society of Chemistry 2020

Supporting Information

Cu_A-Based Chimeric T1 Copper Sites Allow for Independent Modulation of Reorganization Energy and Reduction Potential.

Jonathan Szuster,[a,b] Ulises A. Zitare,[a,b] María A. Castro,[a,b] Alcides J. Leguto,[c,d] Marcos N. Morgada,[c,d] Alejandro J. Vila,[c,d] Daniel H. Murgida[a,b]*

[a]Instituto de Química Física de los Materiales, Medio Ambiente y Energía (INQUIMAE, CONICET-UBA)

[b]Departamento de Química Inorgánica, Analítica y Química-Física, Facultad de Ciencias Exactas y Naturales, Universidad de Buenos Aires, Buenos Aires, Argentina.

[c]Instituto de Biología Molecular y Celular de Rosario (IBR, CONICET-UNR)

[d]Departamento de Química Biológica, Facultad de Ciencias Bioquímicas y Farmacéuticas, Universidad Nacional de Rosario, Rosario, Argentina.

SUPPLEMENTARY FIGURES AND TABLES

Table S1. Reduction potentials and spectroscopic parameters of the chimeras.

	Ami-*Tt*-Cu_A	PAz-*Tt*-Cu_A	Pc-*Tt*-Cu_A	Azu-*Tt*-Cu_A	4A3A-*Tt*-Cu_A	CBP-*Tt*-Cu_A	Rc-*Tt*-Cu_A	NiR-*Tt*-Cu_A	2R2R-*Tt*-Cu_A
$\varepsilon_{450}/\varepsilon_{600}$	1.49	1.28	1.47	1.21	1.10	1.15	1.49	1.64	1.46
ν_{eff}^{Cu-Cys} (cm^{-1})	377	372	377	385	383	359	371	359	371
$E°'$ (mV vs NHE)	419 (±1)	442 (±2)	420 (±4)	470 (±2)	482 (±2)	436 (±1)	428 (±2)	434 (±1)	540 (±1)
$\Delta^{chimera\text{-}wild\ type}E°'$ (mV vs NHE)	+166 (±5)	+210 (±5)	+63 (±5)	+161 (±5)	----	+125 (±5)	-116 (±5)	+194 (±5)	----

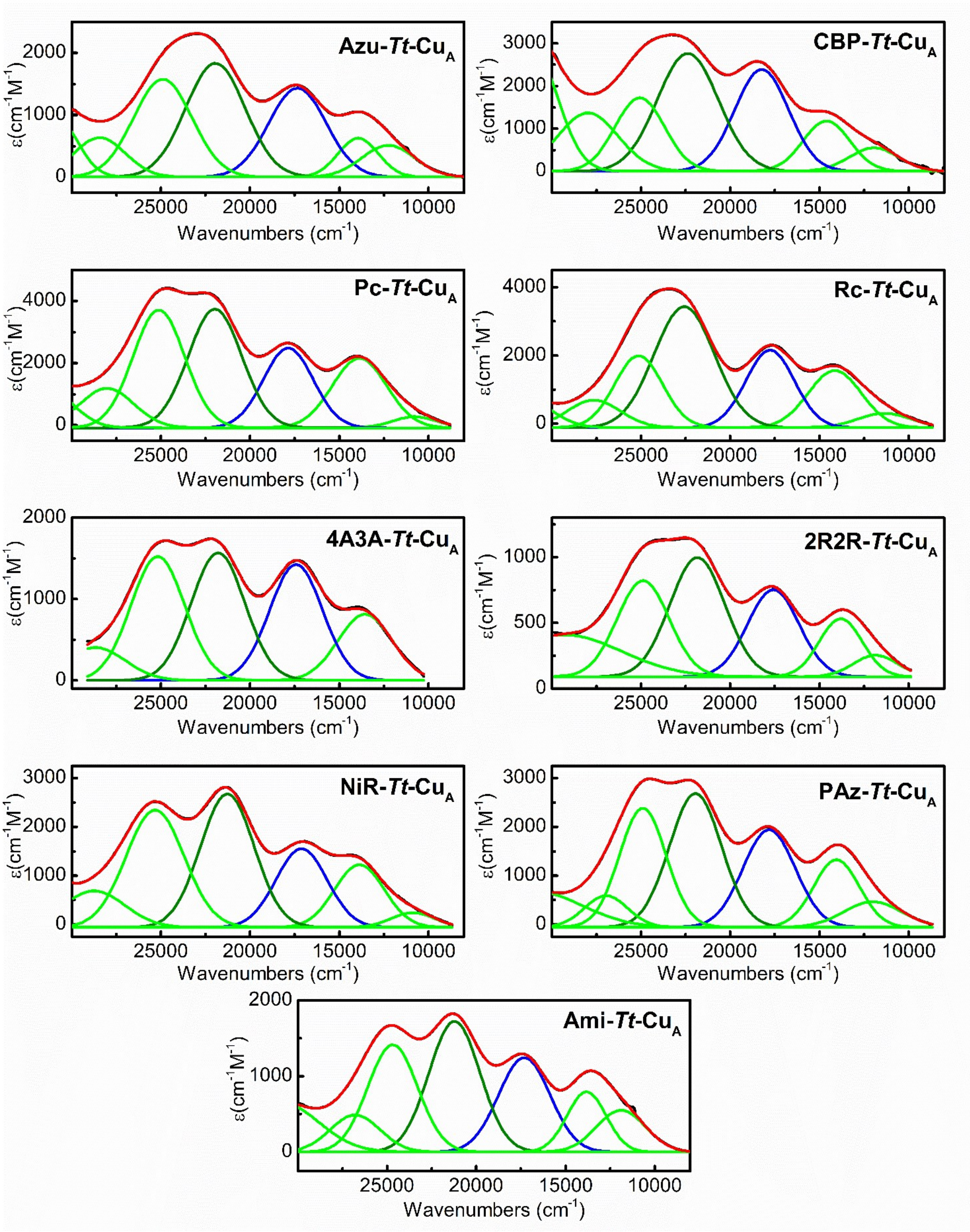

Figure S1. Electronic spectra and the corresponding Gaussian fitting of the chimera are presented. Pseudo-σ $S_{Cys}\rightarrow Cu^{+2}$ and π $S_{Cys}\rightarrow Cu^{+2}$ LMCT transition bands are colored in dark-green and blue, respectively.

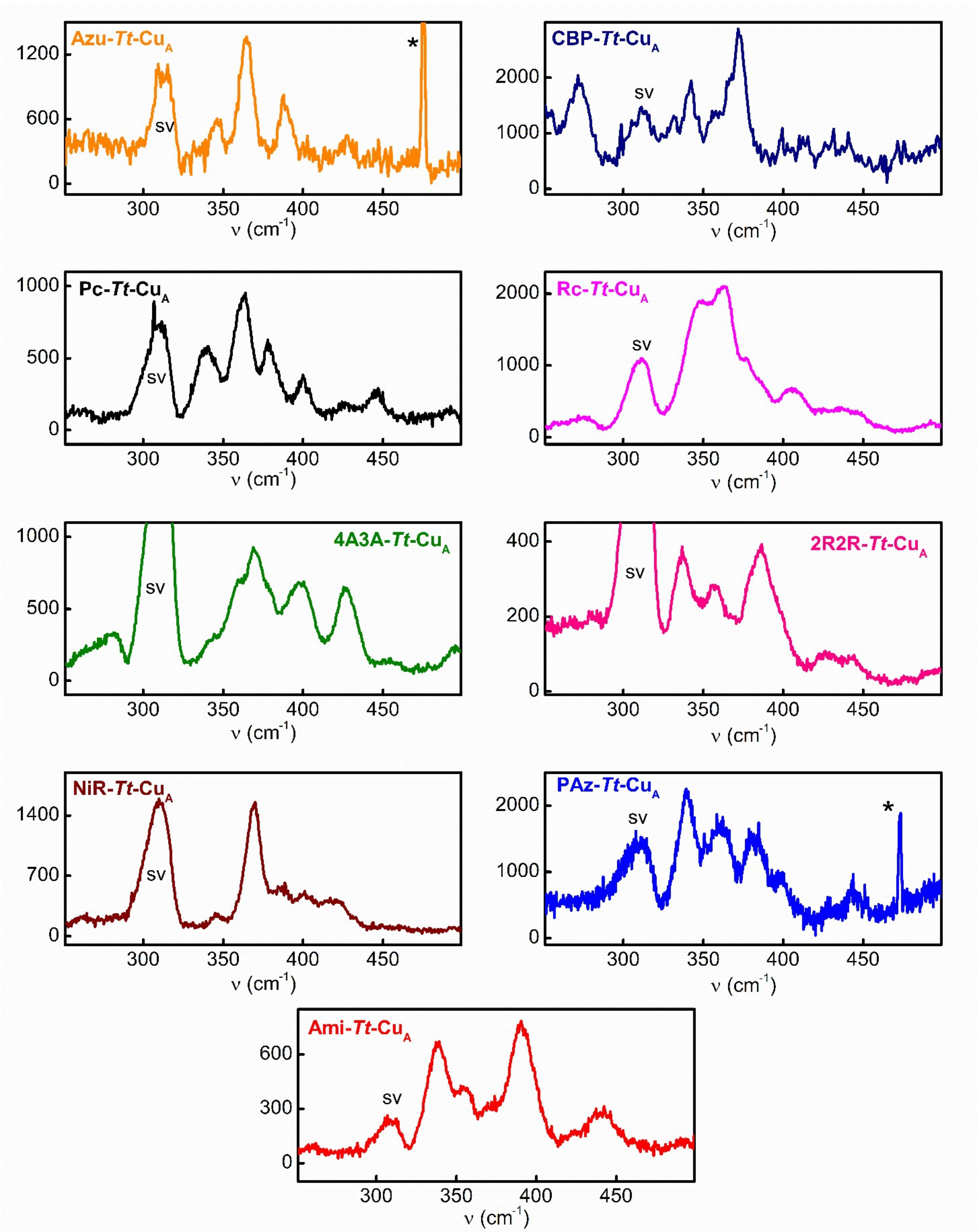

Figure S2. Resonance Raman (rR) spectra of the chimeras in the region of 200-500 cm^{-1} acquired with an 532nm excitation, except for the CBP-*Tt*-Cu$_A$ variant where a 633nm excitation line was employed. Solvent signal (sv) and artifact (*) are labeled when needed.

Table 2. Structural parameters and SASA values for the different chimeras in the oxidized and reduced states obtained by MD simulations.

Oxidized State	Ami-*Tt*-Cu$_A$	PAz-*Tt*-Cu$_A$	Pc-*Tt*-Cu$_A$	Azu-*Tt*-Cu$_A$	4A3A-*Tt*-Cu$_A$	CBP-*Tt*-Cu$_A$	Rc-*Tt*-Cu$_A$	NiR-*Tt*-Cu$_A$	2R2R-*Tt*-Cu$_A$
**Bond Lengths (Å)**									
Cu-S (Cys)	2.217	2.207	2.205	2.230	2.223	2.209	2.221	2.205	2.221
Cu-S (Met)	2.478	2.420	2.434	2.404	2.470	2.415	2.463	2.421	2.472
Cu-N (His-loop)	2.050	2.034	2.030	2.067	2.046	2.039	2.027	2.047	2.040
Cu-N (His-75)	2.023	2.019	2.019	2.003	2.046	2.024	2.029	2.027	2.036
Cu···O (Ile-74)	4.508	4.674	4.648	4.640	4.511	4.576	4.578	4.573	4.640
Cu-S_{Cys}···HN (Gly76)	3,633	3,574	3,611	3,758	3,416	3,456	3,694	3.363	4.007
**Bond Angles (deg.)**									
N(His-75)-Cu -S(Cys-loop)	134.793	138.102	137.817	137.487	134.369	137.325	137.115	133.748	137.251
-N(His-loop)	99.087	97.391	98.472	99.101	99.227	98.359	98.800	99.659	97.973
-S(Met-loop)	90.357	90.710	90.757	98.073	89.463	89.580	88.821	89.023	89.770
S(Cys-loop)-Cu -N(His-loop)	98.032	95.999	96.078	95.361	98.212	95.607	97.432	97.122	98.039
-S(Met-loop)	111.137	110.217	110.081	135.865	111.296	110.240	111.030	110.532	110.644
N(His-loop)-Cu-S(Met-loop)	126.899	129.186	128.108	98.357	127.874	131.046	127.745	131.302	126.968
**Angle between planes (deg.)**									
N_{loop}-Cu-N_{His75} to S_{Cys}-Cu-S_{Met}	62,574	59,560	60,471	57,774	61,899	58,462	60,516	60,053	61,378
**Dihedral angles (deg.)***									
S_{Met}-Cu-S_{Cys}-C_β	-5.429	-0.557	0.008	-23.869	-22.259	-20.992	-12.799	-23.451	13.354 (0.66)<br>-8.920 (0.34)
Cu-S_{Cys}-C_β-C_α	165.151(0.96)<br>-176.288 (0.04)	168.431 (0.87)<br>-175.739 (0.13)	170.163 (0.75)<br>-174.401(0.25)	152.220 (>0.99)	161.141 (0.97)<br>-175.803 (0.03)	164.993 (0.91)<br>-175.397 (0.09)	167.999 (0.93)<br>-176.594 (0.07)	170.396 (0.74)<br>-174.983 (0.26)	167.597 (0.81)<br>-173.734 (0.19)
S_{Cys}-C_β-C_α-N	172.559 (0.39)<br>-171.417(0.61)	171.949 (0.47)<br>-171.663 (0.53)	171.140 (0.55)<br>-172.534(0.45)	175.773 (0.12)<br>-168.065 (0.88)	175.286 (0.15)<br>-167.456 (0.85)	174.988 (0.18)<br>-169.277 (0.82)	174.582 (0.22)<br>-168.818 (0.78)	174.690 (0.24)<br>-170.608 (0.76)	169.849 (0.45)<br>-170.306 (0.55)
**SASA (Å^2)**	442.748	448.167	448.092	474.200	457.935	458.483	458.784	455.983	448.196

*Number in brackets indicate the fraction of the simulation time spent in each value.

Reduced State	Ami-*Tt*-Cu_A	PAz-*Tt*-Cu_A	Pc-*Tt*-Cu_A	Azu-*Tt*-Cu_A	4A3A-*Tt*-Cu_A	CBP-*Tt*-Cu_A	Rc-*Tt*-Cu_A	NiR-*Tt*-Cu_A	2R2R-*Tt*-Cu_A
**Bond Lengths (Å)**									
Cu-S (Cys)	2.355	2.349	2.351	2.361	2.364	2.370	2.370	2.351	2.363
Cu-S (Met)	2.339	2.332	2.316	2.303	2.337	2.332	2.360	2.310	2.356
Cu-N (His-loop)	2.034	2.017	2.018	2.015	2.032	2.014	2.016	2.036	2.033
Cu-N (His-75)	2.058	2.056	2.069	2.063	2.063	2.068	2.058	2.079	2.077
Cu⋯O (Ile-74)	4,579	4,661	4,594	4,573	5,281	4,523	4,544	4.568	4.598
Cu-S_{Cys}⋯HN (Gly76)	3,299	3,329	3,276	3,311	3,263	3,337	3,363	3.318	3.400
**Bond Angles (deg.)**									
N(His-75)-Cu -S(Cys-loop)	111.934	112.441	111.937	112.341	111.973	113.290	113.892	111.870	112.717
-N(His-loop)	102.394	101.567	102.242	105.186	102.555	102.219	100.944	102.407	100.601
-S(Met-loop)	96.829	97.204	98.331	99.497	97.226	96.409	96.360	96.510	96.553
S(Cys-loop)-Cu -N(His-loop)	116.475	116.737	116.625	115.280	116.441	116.246	116.757	115.445	117.354
-S(Met-loop)	112.335	112.221	111.719	111.717	112.505	112.845	113.267	110.472	112.677
N(His-loop)-Cu-S(Met-loop)	114.193	114.010	113.639	111.131	113.502	113.235	112.771	117.674	113.868
**Angle between planes (deg.)**									
N_{loop}-Cu-N_{His75} to S_{Cys}-Cu-S_{Met}	83,606	83,803	84,627	85,728	83,914	83,206	83,160	82.154	83,445
**Dihedral angles (deg.)***									
S_{Met}-Cu-S_{Cys}-C_β	-37.063	-28.462	-28.406	-31.729	-39.588	-34.880	-36.258	-38.230	6.308 (0.12)<br>-17.114 (0.88)
Cu-S_{Cys}-C_β-C_α	175.594 (0.19)<br>-170.455 (0.81)	175.967 (0.08)<br>-166.714 (0.92)	174.537 (0.18)<br>-168.304 (0.82)	174.753 (0.28)<br>-170.816 (0.72)	169.445 (0.75)<br>-173.637 (0.25)	170.179 (0.76)<br>-174.310 (0.24)	168.285 (0.76)<br>-174.335 (0.24)	173.289 (0.06)<br>-160.748 (0.94)	174.08 (0.21)<br>-166.405 (0.79)
S_{Cys}-C_β-C_α-N	176.159 (0.02)<br>-161.188 (0.98)	175.764 (0.06)<br>-163.433 (0.94)	175.098(0.10)<br>-165.478 (0.90)	176.860 (0.04)<br>-165.319 (0.96)	176.351(0.03)<br>-161.114 (0.97)	175.770 (0.09)<br>-164.378 (0.91)	176.069 (0.04)<br>-159.556 (0.96)	175.522 (0.07)<br>-165.092 (0.93)	172.940 (0.33)<br>-169.972 (0.67)
**SASA (Å²)**	443.375	447.096	447.328	462.558	459.544	466.900	465.426	458.119	446.702

*Number in brackets indicate the fraction of the simulation time spent in each value.

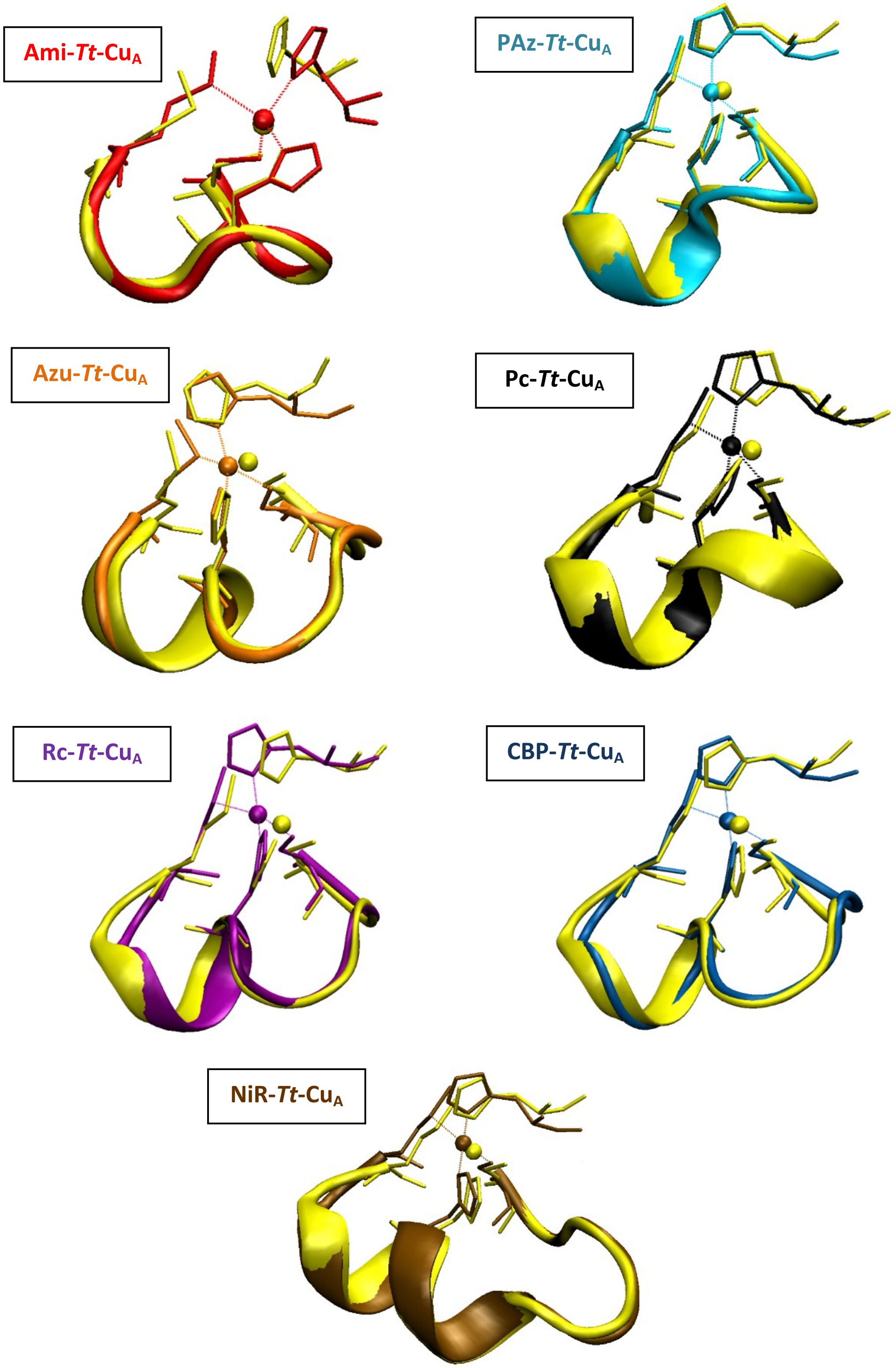

Figure S3. Structural alignment for the metal site (sticks) and loop (new cartoon) moieties are shown. In all cases wild type is colored in yellow, and chimeras follow color code.

Table S3. Structural parameters τ4, Δτ4^{Red-Ox}, and Cu-S(Met)/Cu-S(Cys) distance ratios.

	Ami-*Tt*-Cu$_A$	PAz-*Tt*-Cu$_A$	Pc-*Tt*-Cu$_A$	Azu-*Tt*-Cu$_A$	4A3A-*Tt*-Cu$_A$	CBP-*Tt*-Cu$_A$	Rc-*Tt*-Cu$_A$	NiR-*Tt*-Cu$_A$	2R2R-*Tt*-Cu$_A$
**τ4 parameter Oxidized state**	0.697	0.657	0.667	0.614	0.693	0.649	0.674	0.673	0.679
**τ4 parameter Reduced state**	0.917	0.916	0.920	0.938	0.922	0.925	0.917	0.899	0.913
**Δτ4^{Red-Ox}**	0.220	0.259	0.253	0.324	0.229	0.276	0.243	0.226	0.234
**Cu-S(Met)/Cu-S(Cys) Oxidized**	1.117	1.096	1.103	1.078	1.141	1.093	1.108	1.098	1.113
**Cu-S(Met)/Cu-S(Cys) Reduced**	0.993	0.992	0.984	0.975	0.975	0.983	0.995	0.982	0.997

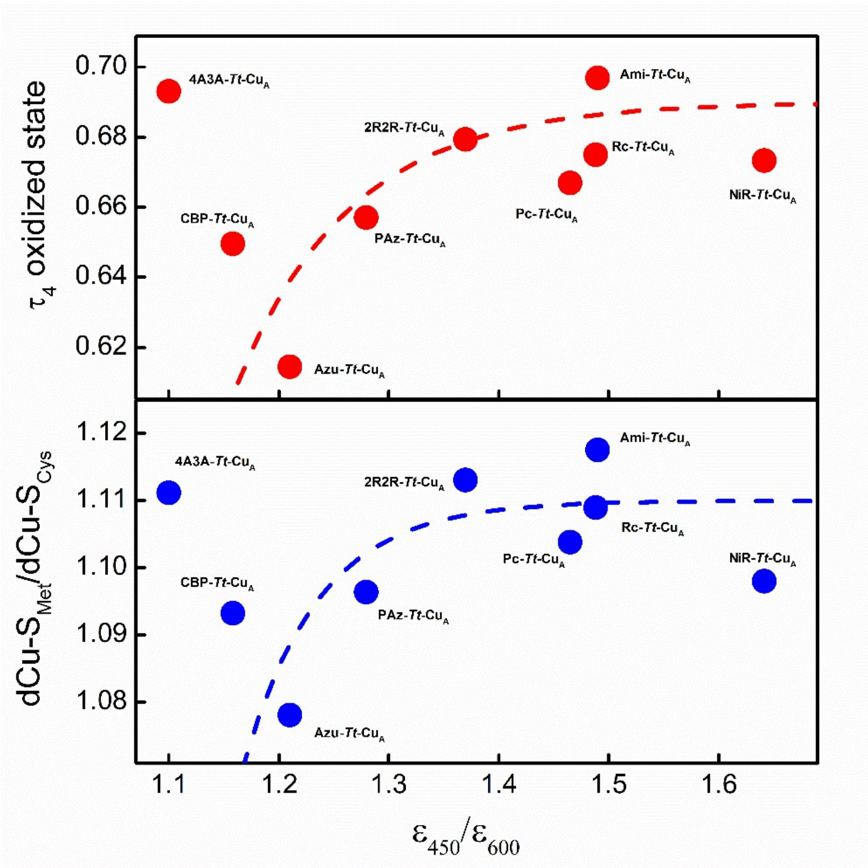

Figure S4. Structural parameters τ4 and Cu-S(Met)/Cu-S(Cys) for the oxidized state plotted against the $\varepsilon_{450}/\varepsilon_{600}$ quotient.

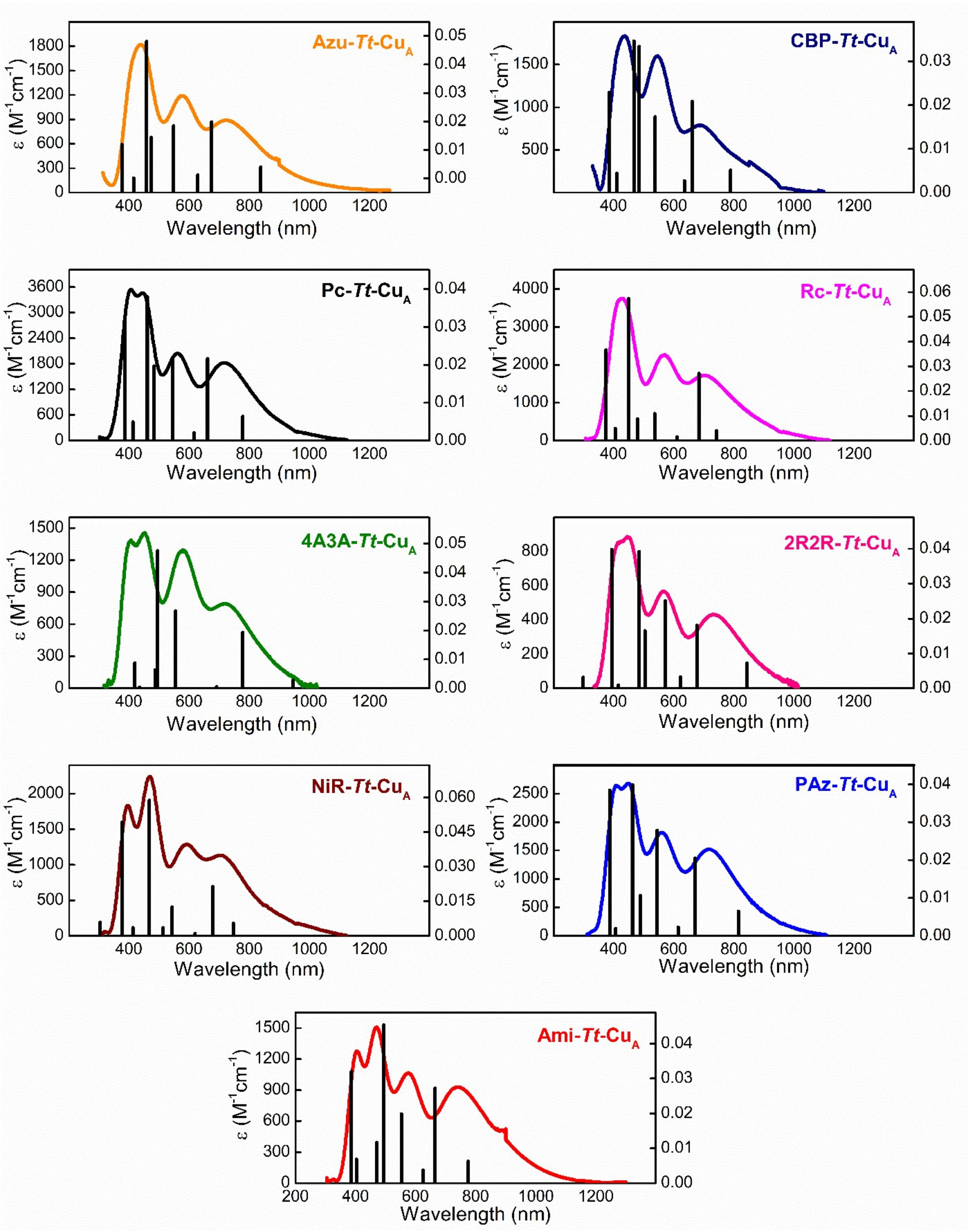

Figure S5. Electronic absorption spectra of the chimeras are presented. Experimental data is plotted following color code and calculated data is plotted in black bar format.

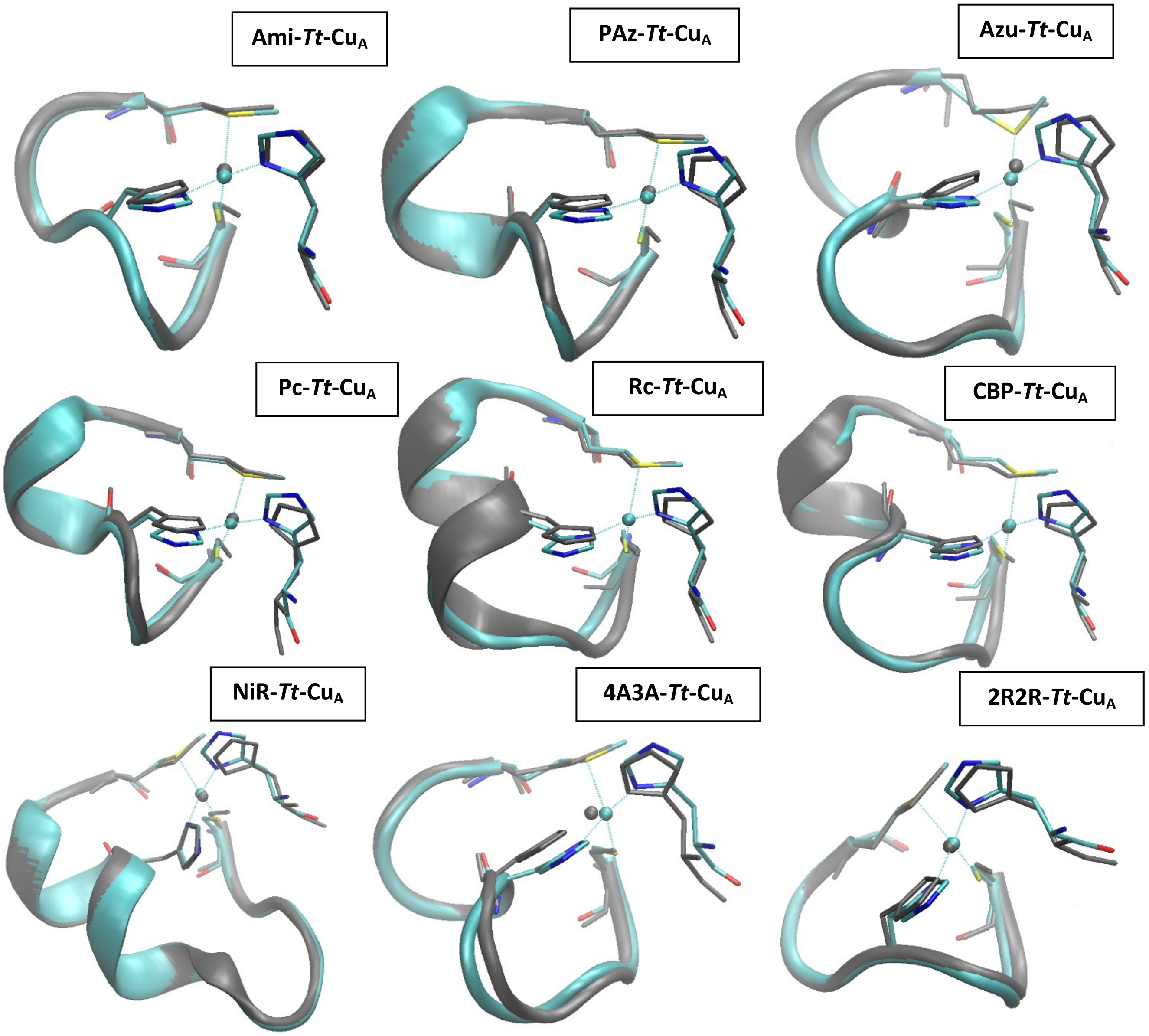

Figure S6. Structural alignment for the metal site (sticks) and loop (new cartoon) moieties for the oxidized (element color code and cyan) and reduced (grey) states. All structures correspond to in silico models obtained as indicated in Computational Methods. The structure of the Ami-*Tt*-Cu$_A$ variant is a snapshot of the MD trajectory obtained using the crystal structure PDB ID 5U7N as starting point.

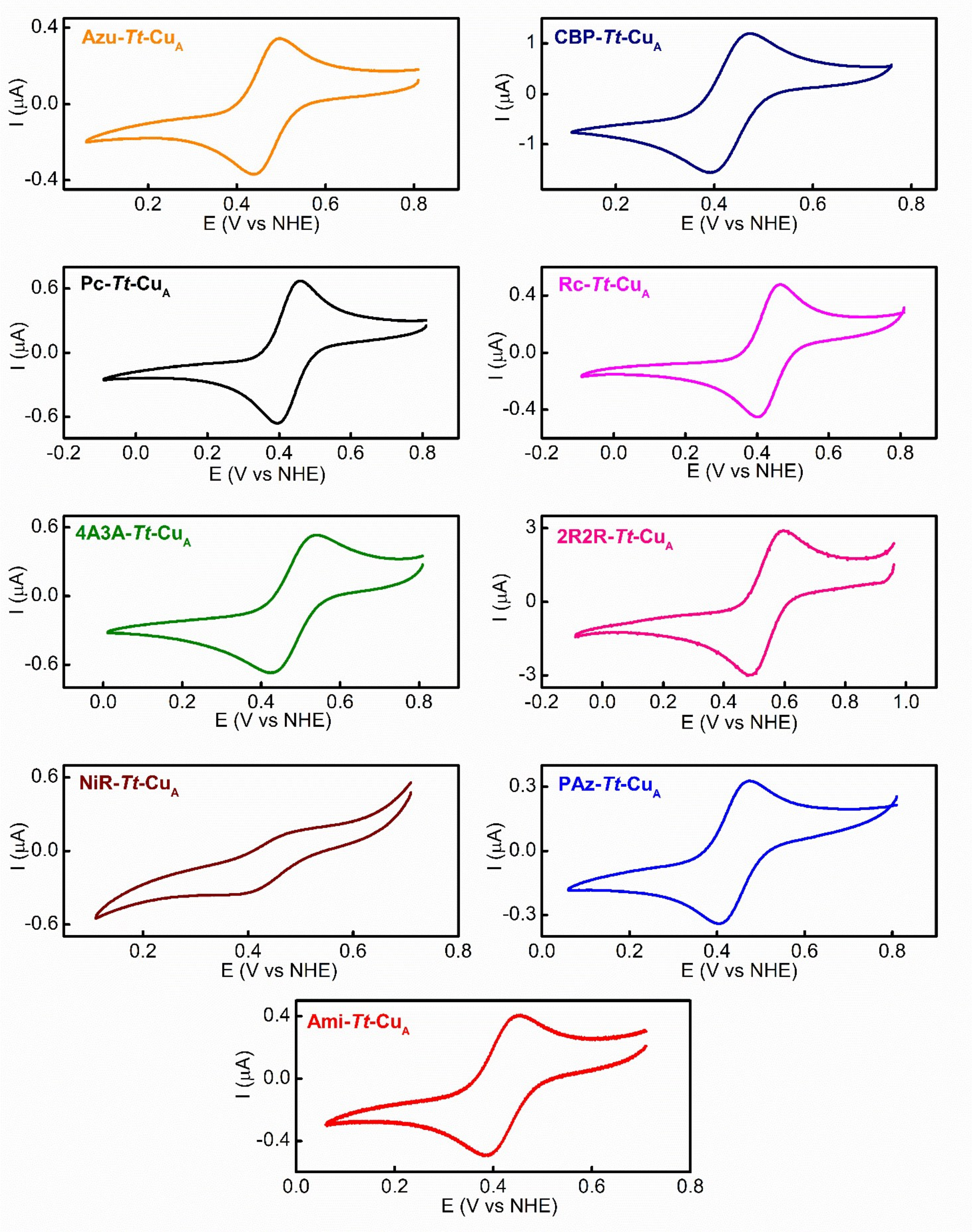

Figure S7. Representative voltammograms of the chimeras obtained from protein solutions. Voltammograms were acquired at 25 °C in 10 mM HEPES buffer (pH 7.0, 500 mM KNO_3) at low scan rates (10 mV/s) to ensure reversibility.

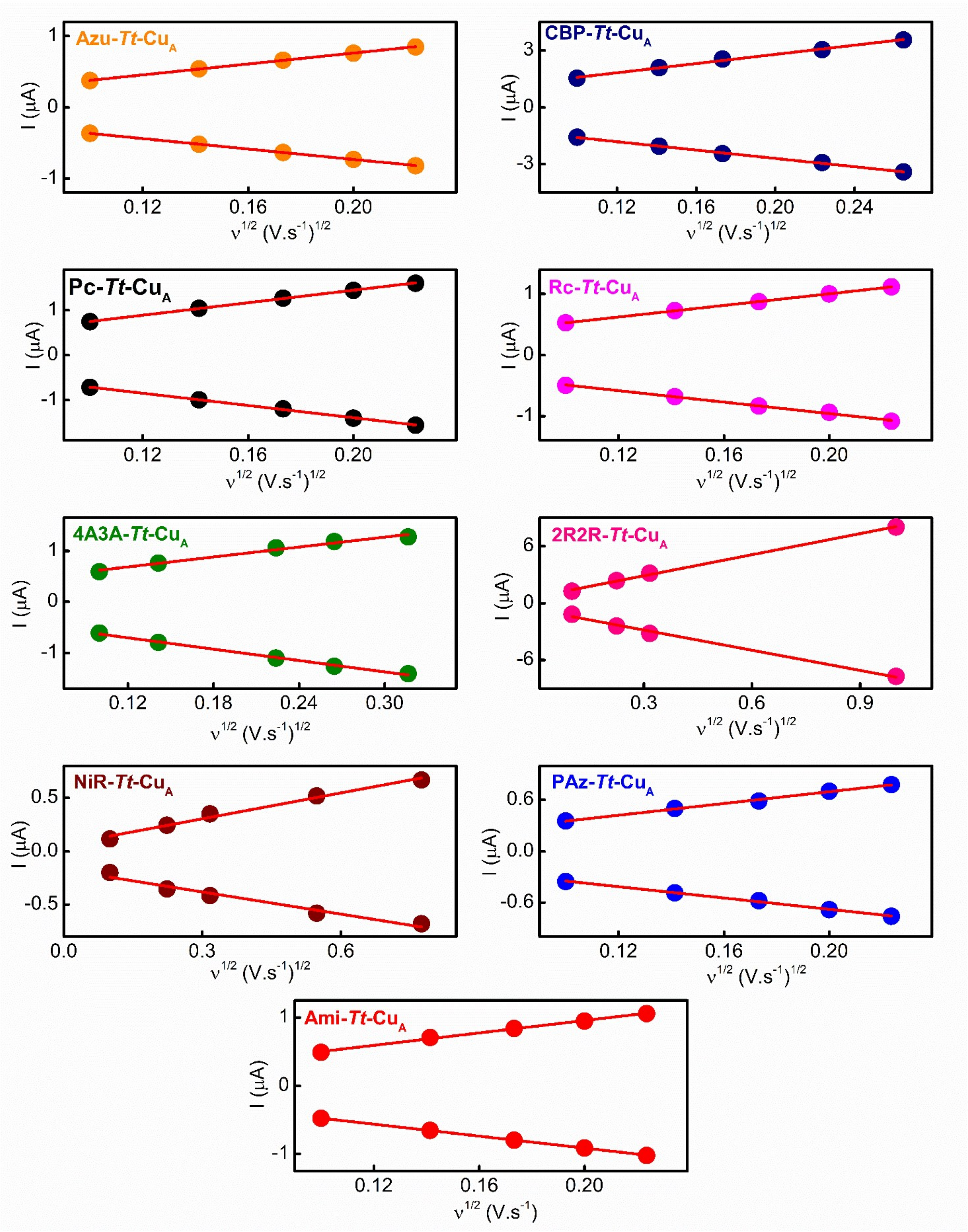

*

Figure S8. Peak currents in function of the square root of the scan rate for cyclic voltammetries of proteins solutions. Linear fits are plotted in red showing a diffusion-controlled one-electron reversible redox process.

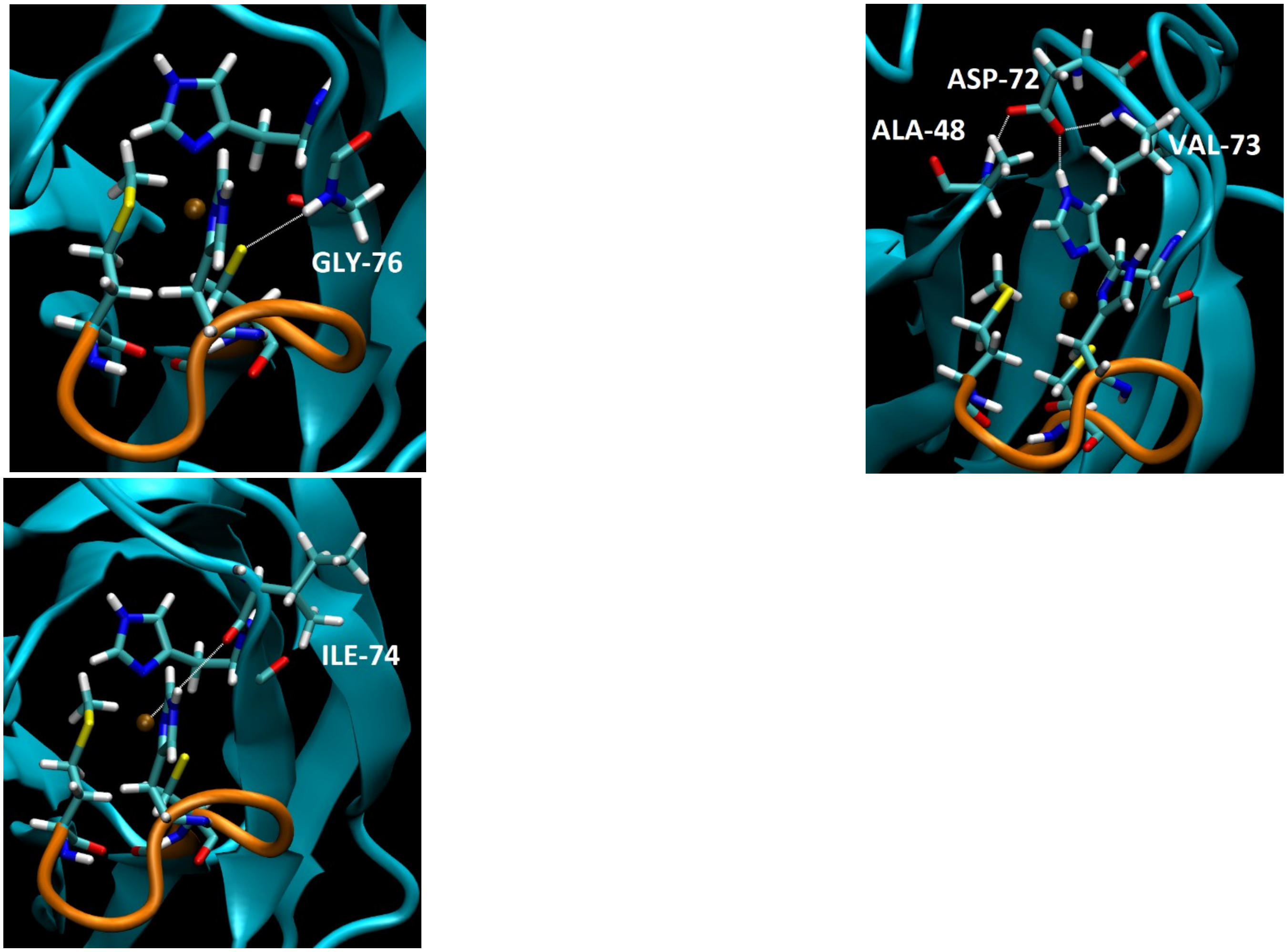

Figure S9. Left: Hydrogen bond from GLY-76 backbone to CYS-110 sulfur. Center: Hydrogen bonding network involving residues HIS-75, ALA-48, ASP-72 and VAL-73. Right: axial backbone carbonyl from ILE-74. The figures are snapshots taken from Ami-*Tt*-Cu$_A$ MD trajectory that uses the crystal structure PDB ID 5U7N as starting point.

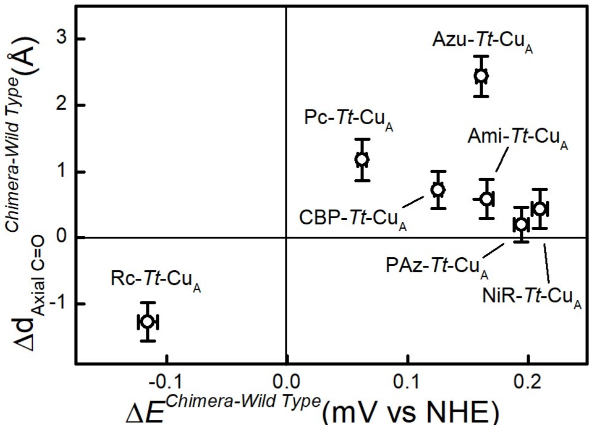

Figure S10. Axial carbonyl distance difference (chimera-wild type) plotted against redox potential difference (chimera-wild type).

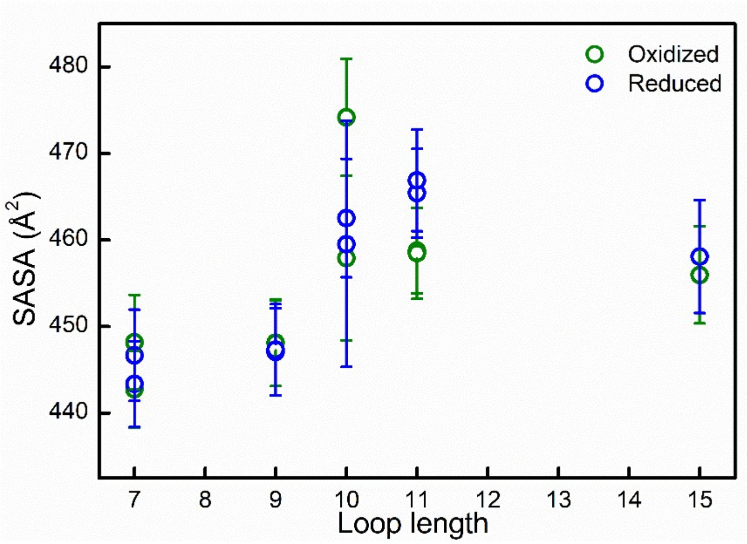

Figure S11. SASA values for the reduced and oxidized states both plotted against the loop length for all the chimeras. SASA error bars represent the standard deviation of the MD trajectory

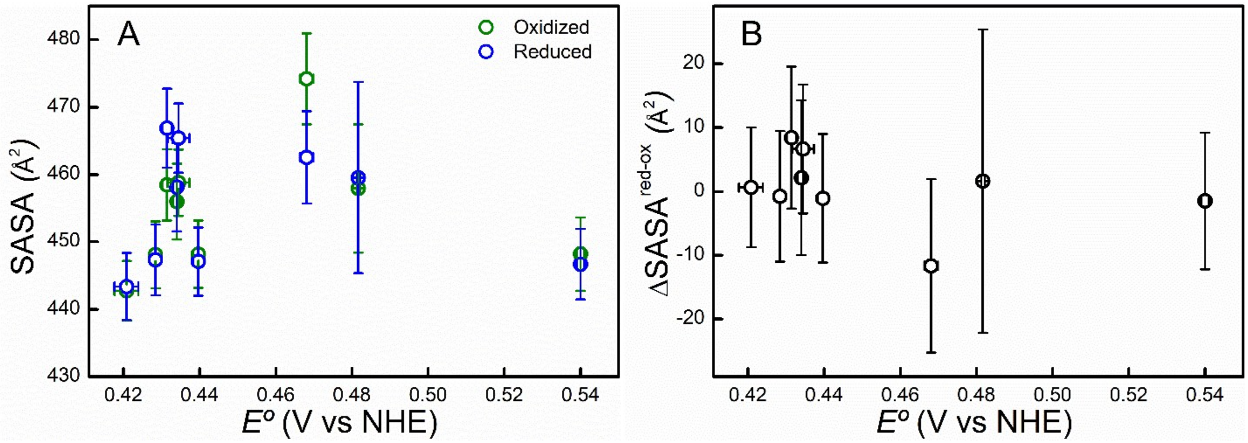

Figure S12. A) SASA values and B) SASA difference between reduced and oxidized states both plotted against reduction potential acquired at 25 °C in 10 mM HEPES buffer (pH 7.0, 500 mM KNO_3). SASA error bars represent the standard deviation of the MD trajectory and the Reduction potential error bars represent the standard deviation of no less than 3 independent experimental measures.

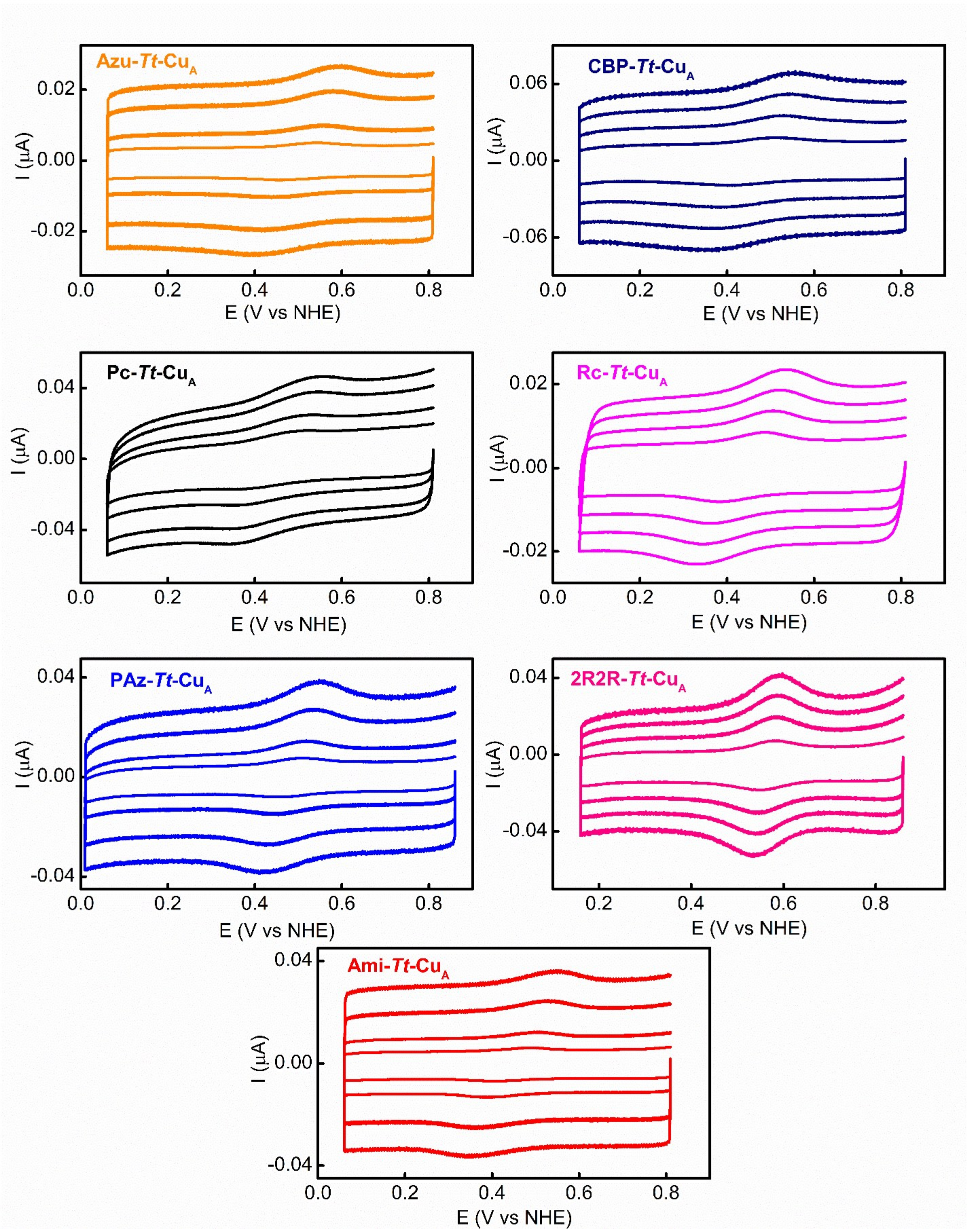

Figure S13. Representative voltammograms of the chimeras obtained from Protein Film Voltammetry experiments. Voltammograms were acquired at 25 °C in 10 mM HEPES buffer (pH 7.0, .250 mM KNO_3). Scan rates were varied from 50 to 500 mV s^{-1} to modify the peak-to-peak separation within the quasi-reversible regime (60-200mV) as required for the Laviron´s method.

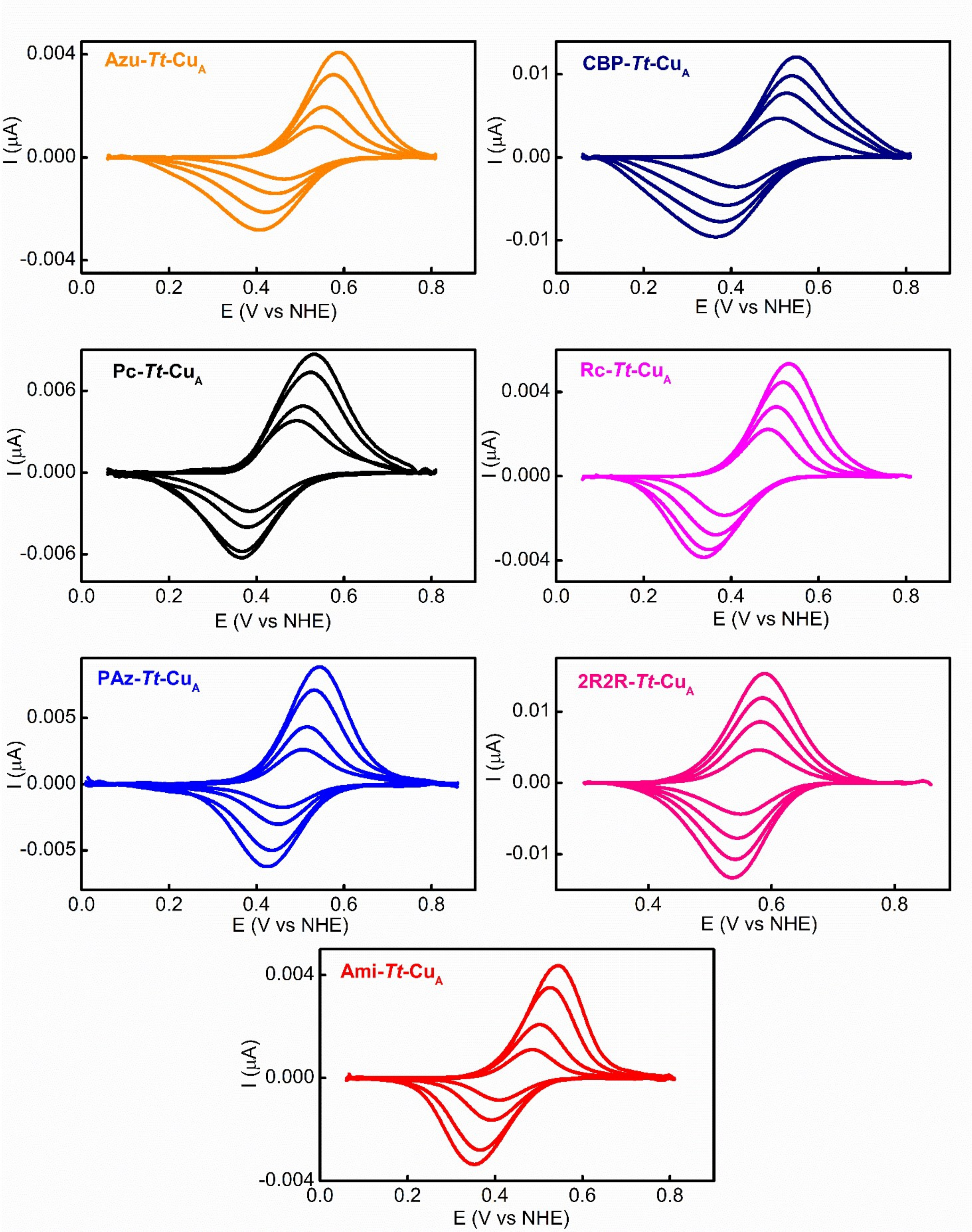

Figure S14. Protein film voltammetries from Figure S13 after substraction of capacitive currents.

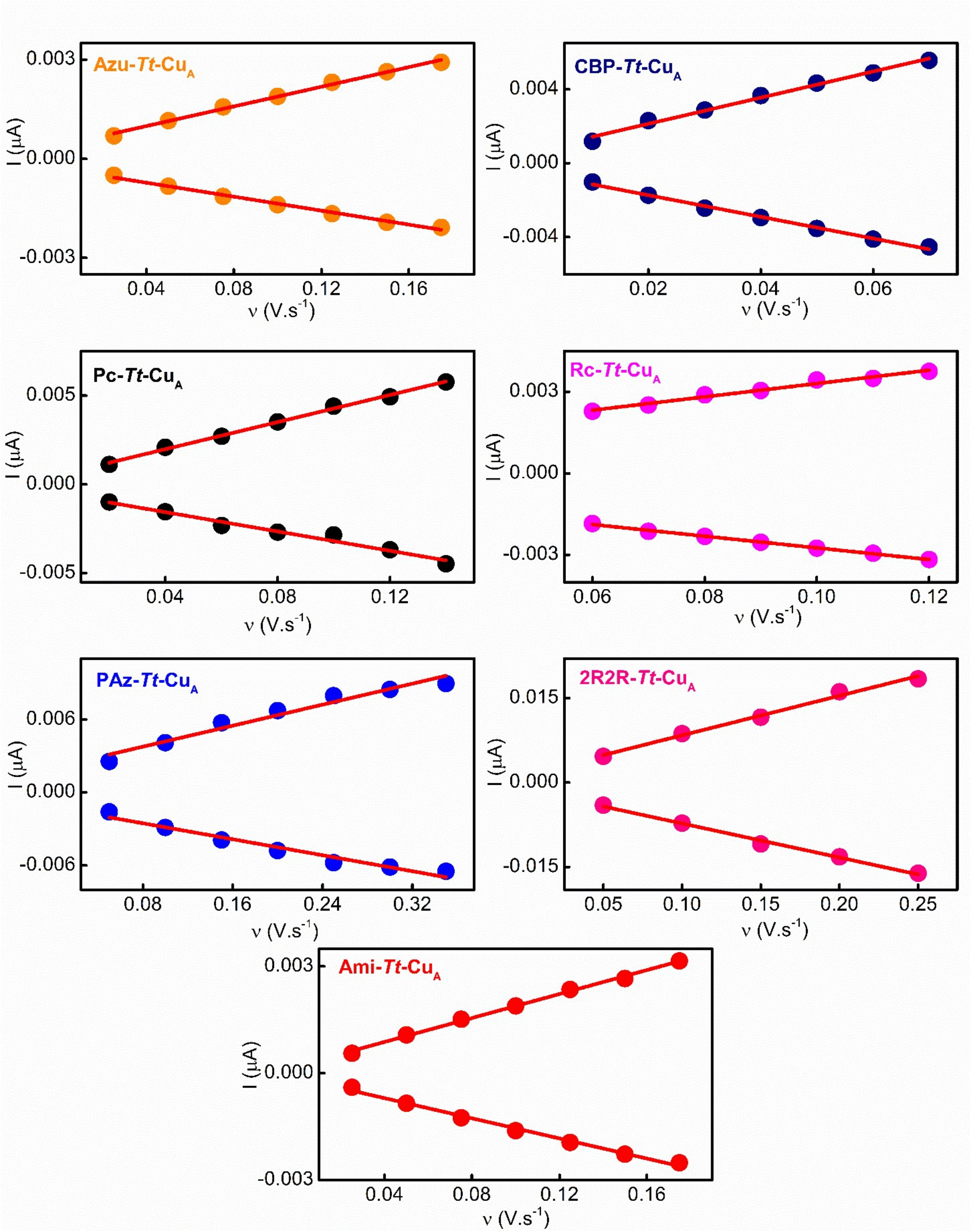

Figure S15. Peak currents as function of the scan rates for Protein Film Voltammetry experiments. Linear fits are plotted in red showing a surface-confined one-electron reversible redox process.

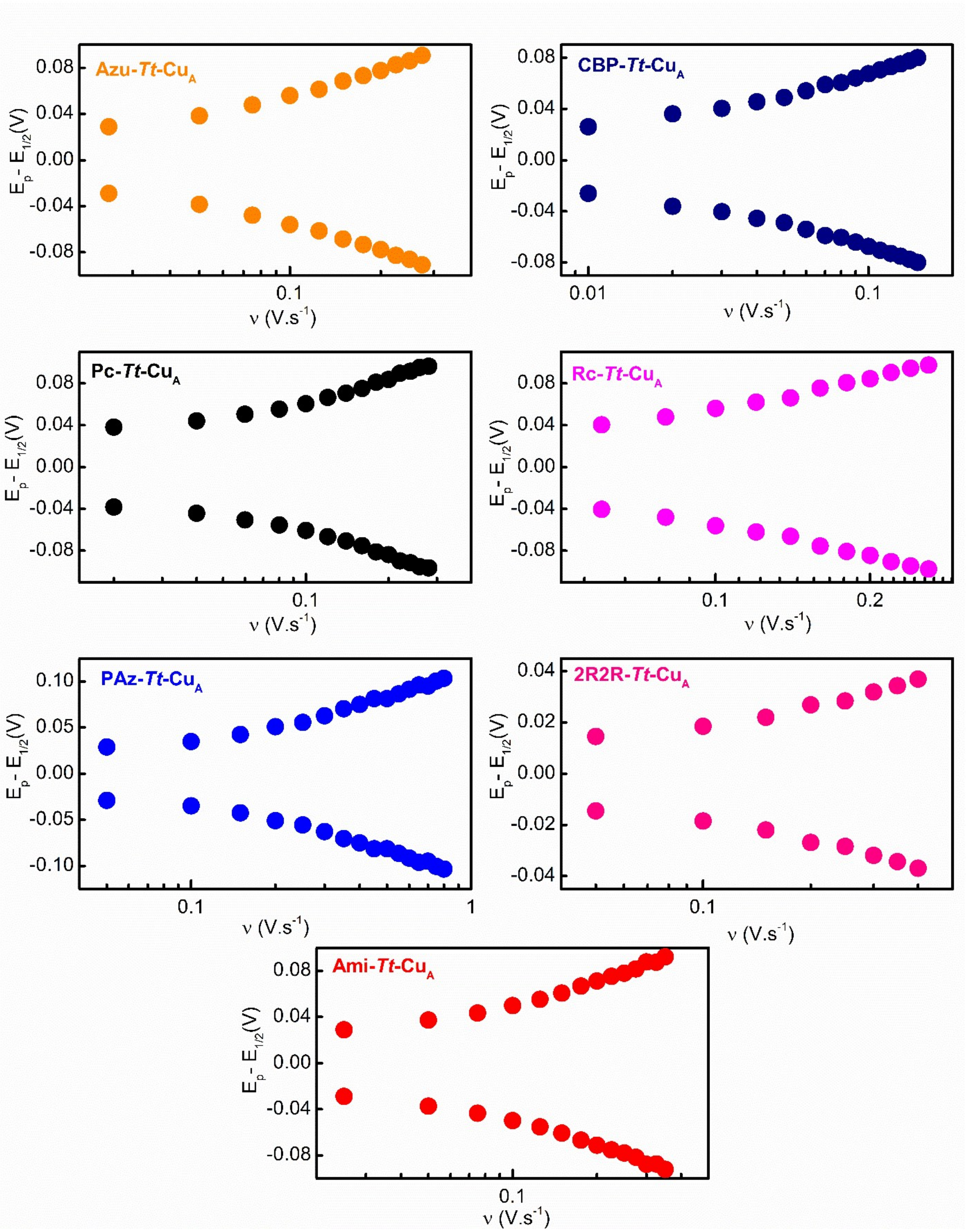

Figure S16. Peak positions (Ep) relative to redox potential ($E_{1/2}$) as a function of the scan rate for Protein Film Voltammetry experiments. Representative data set is shown for measurements acquired at 25°C in 10 mM HEPES buffer (pH 7.0, 250 mM KNO_3) employing Au electrodes coated with self-assembled monolayers (SAMs) of HS-$(CH_2)_{15}$-CH_2OH and HS-$(CH_2)_{15}$-CH_3 in 3:2 ratios. Scan rates were varied from 50 to 500 mV s^{-1} to modify the peak-to-peak separation within the quasi-reversible regime (60-200mV) as required for the Laviron´s method.

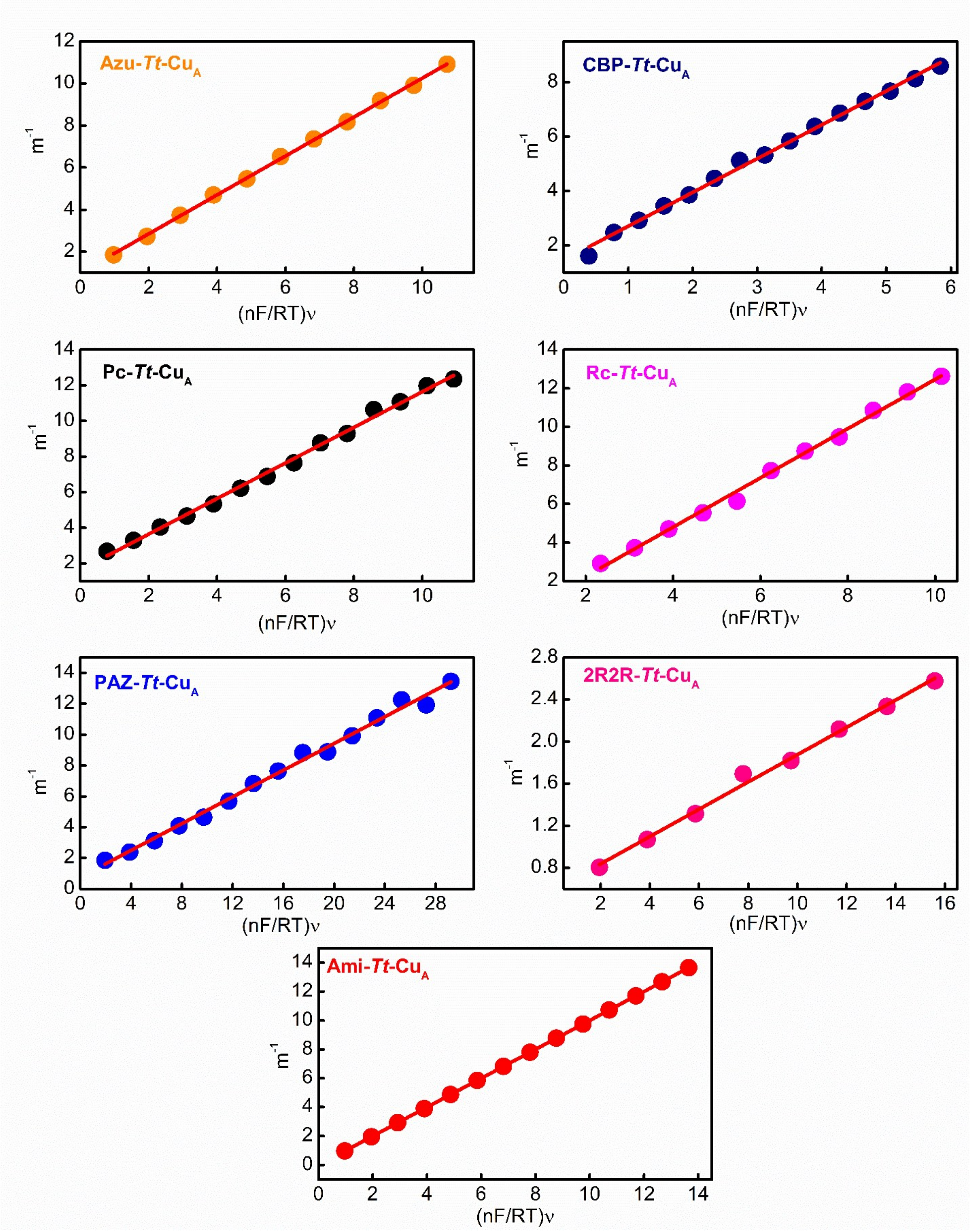

Figure S17. Representative Laviron´s working curves obtained for determining k_{ET}^{0} values for the chimeras adsorbed on Au electrodes coated with self-assembled monolayers (SAMs) of HS-$(CH_2)_{15}$-CH_2OH and HS-$(CH_2)_{15}$-CH_3 in 3:2 ratios. All measures were performed at 25°C in 10 mM HEPES buffer (pH 7.0, 250 mM KNO_3). Scan rates were varied from 50 to 500 mV s^{-1} to modify the peak-to-peak separation within the quasi-reversible regime (60-200mV) as required for the Laviron´s method.

Table S4. Reduction potentials at adsorbed state and Reorganization energies estimated from different methodologies.

	Ami-Tt-Cu_A	PAz-Tt-Cu_A	Pc-Tt-Cu_A	Azu-Tt-Cu_A	CBP-Tt-Cu_A	Rc-Tt-Cu_A	2R2R-Tt-Cu_A
$E°'$ (mV vs NHE)	0.448 (±0.005)	0.484 (±0.005)	0.448 (±0.005)	0.496 (±0.005)	0.457 (±0.005)	0.434 (±0.005)	0.563 (±0.001)
$\lambda_{Exp}^{Arrhenius}$ (eV)	0.29 (±0.05)	0.28 (±0.01)	0.31 (±0.05)	0.47 (±0.04)	0.63 (±0.05)	0.56 (±0.07)	0.3 (±0.1)
λ_{Exp}^{Marcus} (eV)	0.18 (±0.1)	0.17 (±0.1)	0.17 (±0.1)	0.54 (±0.2)	0.69 (±0.2)	0.43 (±0.2)	---

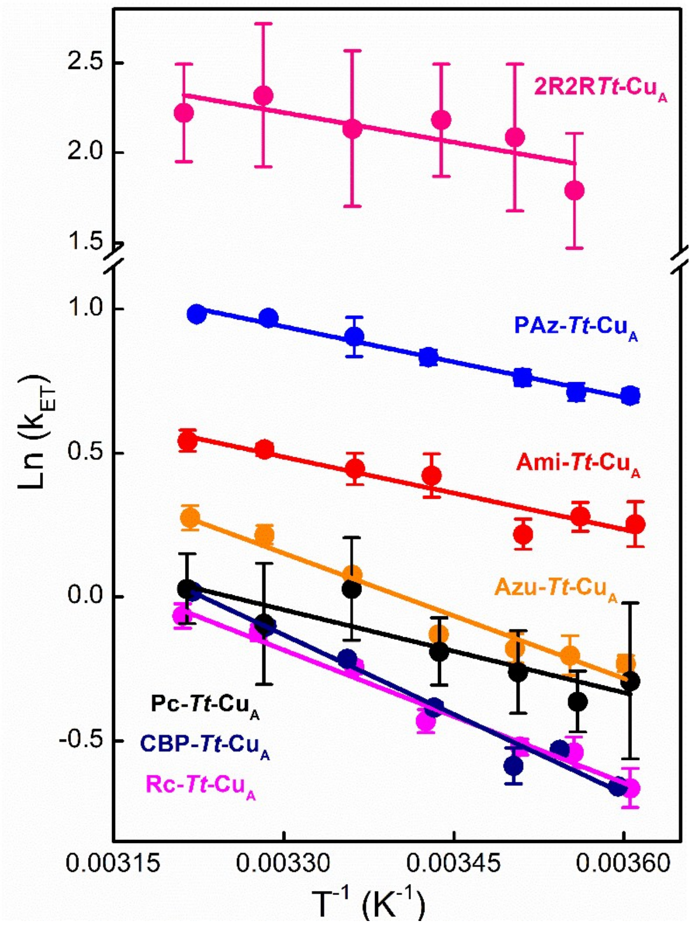

Figure S18. Arrhenius plots obtained from the temperature dependence of k_{ET}^{0} values for the chimeras adsorbed on Au coated electrodes. All measures were performed in the temperature range of 4-40°C in 10 mM HEPES buffer (pH 7.0, 250 mM KNO_3).

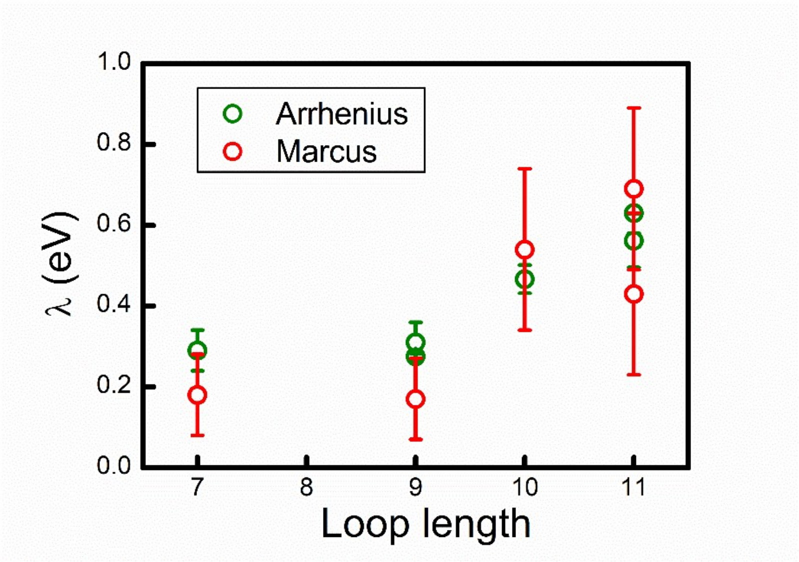

Figure S19. Comparison between Reorganization energies obtained by Arrhenius plot and direct fit of Marcus equation. Both data set show the same trend with the loop length, despite the bigger uncertainty of the second method.

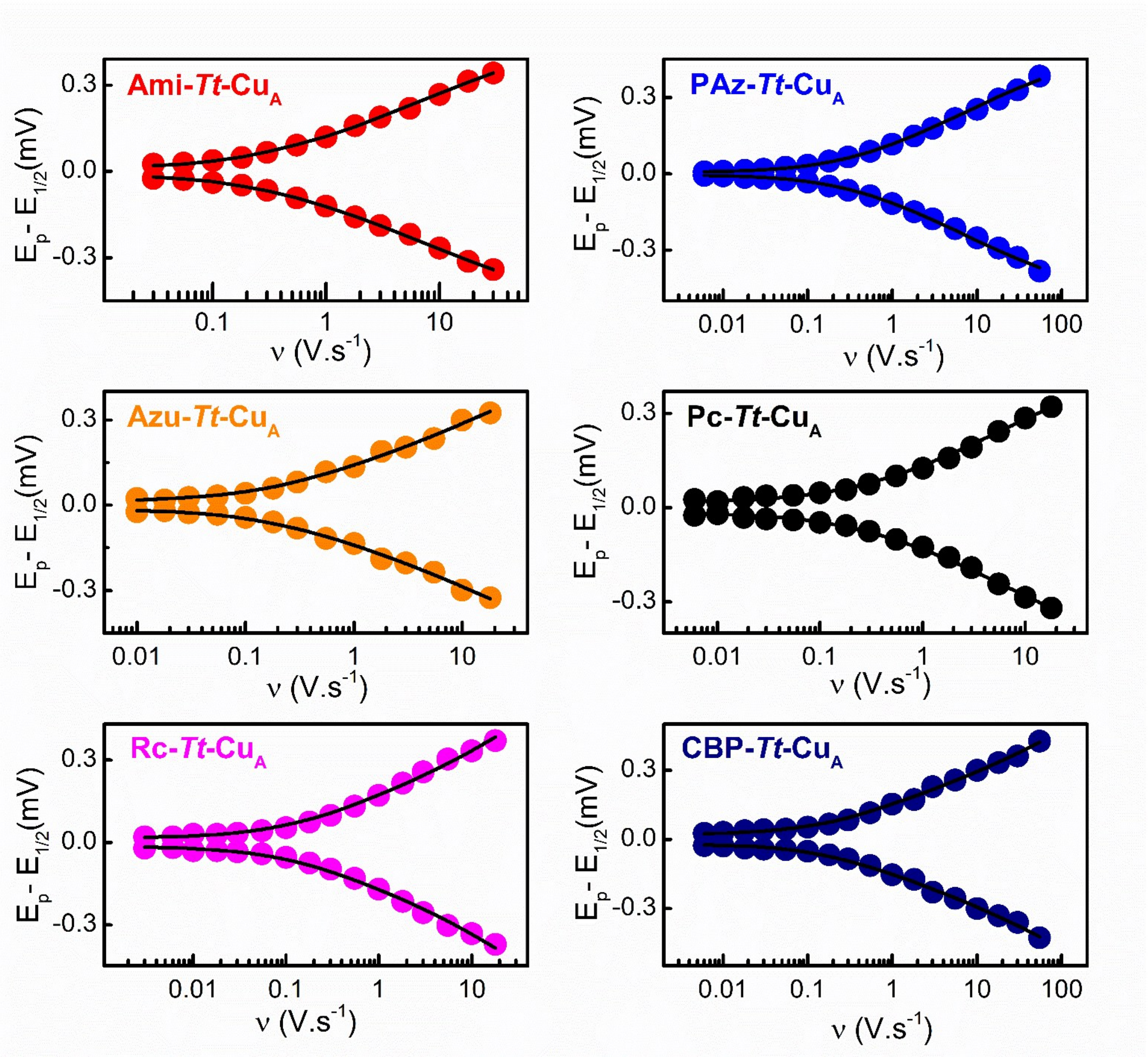

Figure S20. Representative trumpet plots obtained for determining reorganization energies (λ) values by fitting Marcus equation for the chimeras adsorbed on Au electrodes coated with self-assembled monolayers (SAMs) of HS-$(CH_2)_{15}$-CH_2OH and HS-$(CH_2)_{15}$-CH_3 in 3:2 ratios. All measures were performed at 25°C in 10 mM HEPES buffer (pH 7.0, 250 mM KNO_3). Fittings (shown in black) were obtained using a homemade software. Scan rates were varied up to 70 V s^{-1} to achieve sufficiently high peak-to-peak separations (well above 200 mV) that allow for reliable fittings.

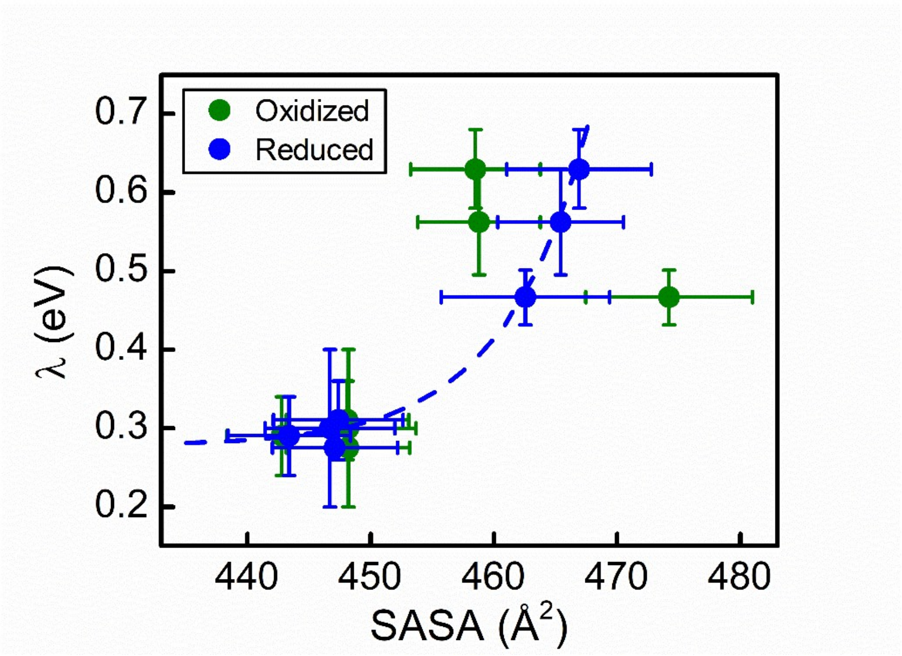

Figure S21. Correlation of SASA (both for oxidized and reduced states) with the reorganization energy for the entire set of chimeras. Error bars for SASA values represent de standard deviation of the MD trajectories, while for the lambda values represent standard deviations of no less than 3 independent measures.

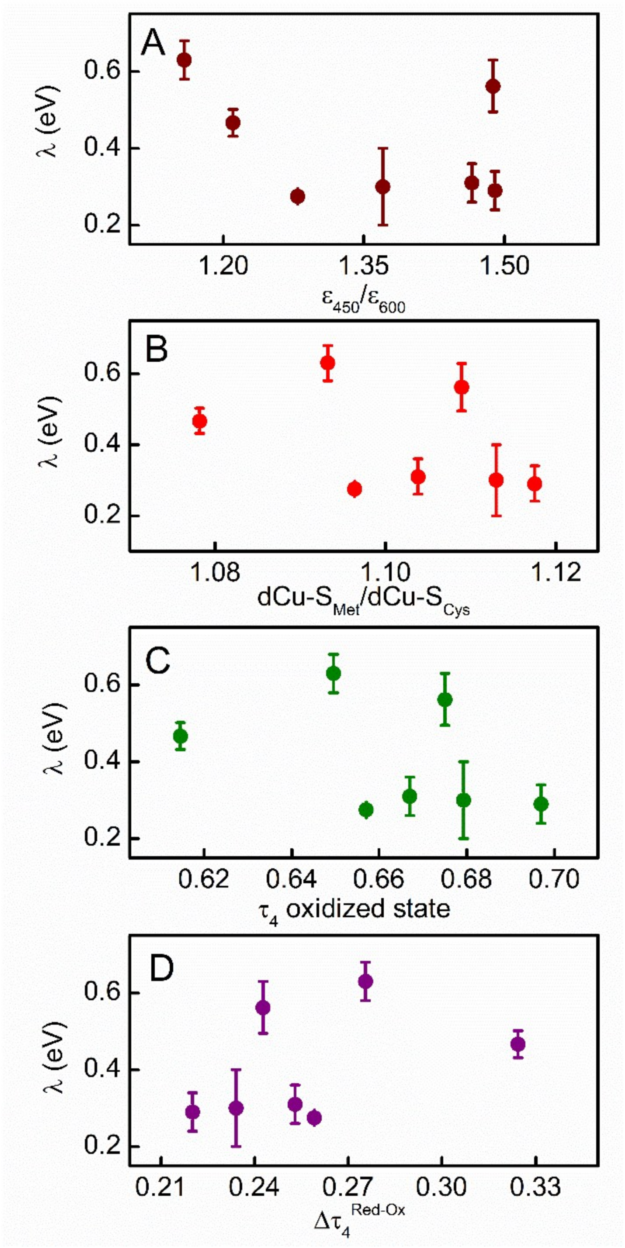

Figure S22. Reorganization energies plotted against (A) ε450/ε600 ratio, (B) dCu-S(Met)/dCu-S(Cys) ratio, (C) τ4 parameter and (D) the difference of τ4 for reduced and oxidized states.
